# Root-enriched β-amylase *GmBAM-like 1* enhances drought and salt tolerance in Arabidopsis

**DOI:** 10.1590/1678-4685-GMB-2025-0234

**Published:** 2026-08-14

**Authors:** Fábia Guimarães-Dias, Lucas Leal Lima, Anna Cristina Neves-Borges, João Travassos-Lins, Dulce Mantuano, Michel Georges Alberts Vincentz, Américo José Carvalho Viana, Márcio Alves-Ferreira

**Affiliations:** 1 Universidade de São Paulo (USP), Escola de Engenharia de Lorena (EEL), Departamento de Biotecnologia, Lorena, SP, Brazil.; 2 Universidade Federal do Rio de Janeiro (UFRJ), Departamento de Genética, Rio de Janeiro, RJ, Brazil.; 3 Universidade Federal do Estado do Rio de Janeiro (UNIRIO), Departamento de Botânica, Rio de Janeiro, RJ, Brazil.; 4 Universidade Federal do Rio de Janeiro (UFRJ), Departamento de Botânica, Rio de Janeiro, RJ, Brazil.; 5 Universidade Estadual de Campinas (Unicamp), Departamento de Biologia Vegetal, Campinas, SP, Brazil.

**Keywords:** Abiotic stress, beta amylase gene, drought stress, gene expression

## Abstract

Water scarcity impacts soybean cultivation and productivity globally. The ability of plants to withstand drought stress involves complex molecular and physiological mechanisms that facilitate the restoration and maintenance of cellular homeostasis. This study identified genes associated with carbohydrate metabolism and GABA shunt pathway that respond to water deficit in two soybean varieties. These varieties exhibited contrasting responses to water scarcity, and were subjected to two distinct cropping systems. In the drought-tolerant variety, a strategy for conferring tolerance was observed through the pre-emptive priming of the drought response. By applying multivariate analysis, we identified a pivotal gene, *GmBAM-like 1*, which responds to water scarcity. *GmBAM-like 1* encodes a β-amylase and showed rapid activation and elevated expression levels in root tissues of the tolerant variety, suggesting its potential involvement in the drought tolerance response. Transgenic Arabidopsis plants overexpressing *GmBAM-like 1* demonstrated enhanced tolerance to salt and osmotic stress, as evidenced by increased survival and germination rates. Additionally, after drought stress, these plants showed higher transpiration rates, larger leaf area, and greater relative water content upon rehydration. These findings demonstrate the potential of integrating the *GmBAM-like 1* gene into plant breeding programs to develop cultivars with improved tolerance to water, salt, and osmotic stresses.

## Introduction

Soybean (*Glycine max* L. Merr), a key legume native to Asia, holds immense economic value due to its diverse applications in animal feed, human food, pharmaceuticals, and biodiesel production (Mishra et al., 2024). For the 2023/2024 marketing year, global soybean production is estimated at 362.31 million metric tons, with Brazil and the United States contributing nearly 70% of this total (Valdes et al., 2023). However, drought stress remains a critical limiting factor affecting soybean yield and production, with significant losses reported during reproductive stages (Felisberto et al., 2023). Understanding soybean’s drought tolerance mechanisms and identifying the underlying genes and metabolic pathways are crucial for mitigating these losses.

Drought stress triggers extensive metabolic reprogramming in soybean and various other plants, leading to altered gene expression in pathways responsible for the synthesis of protective, regulatory, signaling, and structural molecules (Sun et al., 2022; Wang et al., 2024). Osmoprotectants and osmolytes, including soluble carbohydrates, proteins, free amino acids, glycine betaine, and proline, play a significant role in protecting membranes and proteins from the denaturing effects of oxidative stress while maintaining cell turgor and mitigating dehydration (Ghosh et al., 2022). Conversely, signaling molecules like γ-Aminobutyric acid (GABA) function primarily by coordinating biochemical changes that regulate various physiological processes essential for adaptation to water scarcity (Rajasheker et al., 2019; Singh et al., 2019; Tiwari et al., 2021; Chen et al., 2022). GABA shows rapid accumulation in plant tissues as a response to various stresses, including drought. Its synthesis involves the GABA shunt pathway, where glutamic acid decarboxylase (GAD) catalyzes the irreversible α-decarboxylation of glutamate to GABA (Hasan et al., 2021). Drought-induced GABA accumulation offers cellular protection by stimulating the synthesis of osmoprotectants, soluble sugars, and proline while facilitating osmotic regulation (Ozturk et al., 2021). It additionally influences stomatal opening regulation, enhances antioxidant enzyme activity, promotes nitrogen recycling, protects photosystem II, regulates wax biosynthesis and fatty acid oxidation, delays leaf senescence, and contributes to cell wall biosynthesis (Chen *et al*., 2022).

Water scarcity also significantly disrupts carbohydrate metabolism pathways (J. Wang et al., 2022). These pathways provide precursors for osmoprotectants, osmolytes, and signaling molecules and play a crucial role in energy production (Shiade et al., 2024). Drought conditions substantially decrease photosynthetic efficiency due to stomatal closure and impaired electron flow, reducing carbon assimilation and ATP and NADPH production (Ahluwalia et al., 2021). Critical pathways in carbohydrate metabolism compensate for the decreased photosynthetic activity by supplying carbon skeletons and energy for downstream biosynthetic processes (AbdElgawad et al., 2020). 

β-Amylases (BAMs) (EC 3.2.1.2) are enzymes involved in carbohydrate metabolism, acting as endo-acting glucan hydrolases. They specifically cleave α-1,4-glycosidic bonds in starch, releasing maltose units from the non-reducing end of the polysaccharide chain (Fan et al., 2024). BAMs regulate gene expression and various physiological processes, including growth, development, defense responses, and abiotic stress responses (Yue et al., 2019; Ma et al., 2022). In Arabidopsis, BAM1 degrades starch in guard cells, regulating stomatal opening during the day. Additionally, BAM1 contributes to starch turnover in mesophyll cells under drought or salt stress (Thalmann and Santelia, 2017; Thalmann *et al*., 2019). Researchers demonstrated improved plant performance under drought stress in Arabidopsis *bam1* mutants when they inhibited BAM1-mediated starch breakdown in guard cells (Prasch et al., 2015). 

In this study, we investigated the expression profile of 13 genes associated with carbohydrate metabolism and the GABA shunt pathway in two contrasting soybean varieties grown under drought stress in hydroponics and pot-based systems. *GmBAM1-like 1* (*Glyma.09G168300.1*) was chosen for further *in vivo* functional analysis in a heterologous Arabidopsis system due to its high expression and induction in the tolerant cultivar’s roots. *GmBAM-like 1* transgenic Arabidopsis displayed a higher rate of starch degradation and significantly enhanced drought and salt tolerance.

## Material and Methods

### Plant material and cultivation systems

Leaves and roots were collected from the soybean genotypes Embrapa 48 and BR16 grown in both a pot-based system (PSys) and a hydroponic system (HSys), following established protocols (Guimarães-Dias et al., 2012; Neves-Borges et al., 2012). The HSys used 30 liters plastic containers (Figure S1) with an aerated nutrient solution (pH 6.6), as described previously (Martins et al., 2008). At vegetative stage V4, characterized by four fully expanded trifoliolate leaves with distinct petioles and stipules, plants were acclimated and then subjected to rapid stress induction in their respective systems. In the PSys, leaves and roots from both genotypes were harvested after seven days (Ψw = −1.5 ± 2.0 MPa; moderate stress) and after ten days (Ψw = −3.0 ± 2.0 MPa; severe stress). In the HSys, leaves and roots were harvested after removing plants from the hydroponic solution and placing them on trays for 50, 100, and 150 minutes to simulate drought stress by air drying. Two independent biological replicates were collected for each experimental condition and used for subsequent relative-expression analyses.

### *In silico* identification of putative drought-responsive genes in soybean

An *in silico* analysis, adapted from Guimarães-Dias et al. (2012), was carried out to identify soybean genes potentially involved in drought responses. First, a comprehensive literature review identified Arabidopsis genes implicated in drought stress pathways (Sanchez et al., 2008; Bundy et al., 2009; Urano et al., 2009; Hey et al., 2010). The AraCyc metabolic pathway database (Zhang P. et al., 2005) and KEGG pathway tools (Zhang J. D. and Wiemann, 2009) were used to select specific drought-responsive pathways and genes in Arabidopsis. Second, the putative soybean homologs of these Arabidopsis genes were identified using Phytozome. We applied stringent criteria (preferably e-value = 0); when perfect matches were not available, the top five hits with e-values < 10⁻³⁰ were retained for further analysis. The putative soybean orthologs were evaluated using the platform described by Rodrigues et al. (2012), which includes Solexa cDNA subtractive libraries from twenty-two soybean cultivars exposed to various drought treatments and time courses. Finally, a two-step refinement process was applied to select *GmBAM-like 1* as a target gene, comprising: (i) expression analysis (RT-qPCR); and (ii) multivariate analyses (hierarchical clustering heatmap, principal component analysis (PCA), and Random Forest). Detailed procedures for these steps are provided below.

### Phylogenetic analysis of β-amylases from diverse plant species

β-amylase protein sequences were retrieved from the proteomes of eight plant species available in Phytozome: *Solanum lycopersicum* (ITAG4.0), *Vitis vinifera* (v2.1), *Glycine max* (Wm82.a4.v1), *Populus trichocarpa* (v4.1), *Arabidopsis thaliana* (Araport11), *Ananas comosus* (v3), *Oryza sativa* (v7.0), and *Setaria viridis* (v4.1). These species were chosen to represent broad angiosperm diversity. The HMM profile for the Pfam Glycosyl hydrolase family 14 domain (PF01373) was obtained from InterPro and used with HMMER 3.3.2 (http://hmmer.org) to identify candidate β-amylases. Candidate sequences had their domains annotated and confirmed using InterProScan (Jones et al., 2014). Confirmed full-length sequences were aligned with MUSCLE (implemented in MEGA X; Kumar et al., 2018) using default gap penalties. The ModelFinder tool (IQ-TREE v1.6.12) was used to select the optimal substitution model (JTT+F+R7) based on the Bayesian information criterion (Jones *et al.*, 1992; Soubrier et al., 2012; Trifinopoulos et al., 2016; Minh et al., 2020). Maximum-likelihood inference was performed with IQ-TREE, and branch support was estimated by UFBoot with 2,000 bootstrap replicates (Hoang et al., 2018). The resulting tree was midpoint rooted.

### Quantification of transcript levels in drought-stressed plants

Total RNA was extracted from leaf and root samples of PSys plants using the Plant RNeasy Kit (Qiagen). Genomic DNA was removed by on-column DNase I (RNase-free) digestion (Biolabs). RNA concentration and purity were assessed by A260/A280 ratios on a NanoDrop™ ND-1000 spectrophotometer (Thermo Scientific), and integrity was verified by 1% agarose gel electrophoresis. For leaf samples, cDNA was synthesized from 1 µg total RNA using 50 µM oligo(dT) and 10 µM of each dNTP. After incubation at 65 °C for 5 min and chilling on ice, 20 mM DTT, 1× First-Strand Buffer, and 200 U SuperScript™ III reverse transcriptase (Invitrogen) were added. Reverse transcription proceeded at 50 °C for 1 h, followed by inactivation at 70 °C for 15 min; cDNA was stored at −20 °C. Root RNA samples were analyzed using the Power SYBR^®^ Green RNA-to-Ct™ One-Step Kit (Applied Biosystems) on an Eppendorf Realplex/ Mastercycler Epgradient sequence-detection system, following the manufacturer’s instructions. Reactions (20 µL) contained 25 ng RNA and were run in technical triplicates with a negative control. Cycling: 48 °C for 30 min (reverse transcription), 95 °C for 10 min (denaturation), then 40 cycles of 95 °C for 15 s and 60 °C for 1 min with fluorescence acquisition. A melting-curve analysis was performed at the end. Leaf cDNA samples were analyzed in 96-well optical plates on an ABI 7500 Fast Real-Time PCR system (Applied Biosystems) using SYBR^®^ Green. Each 20 µL reaction contained 10 µL diluted cDNA (1:50), 0.2 µM of each primer (Table S1), 50 µM dNTPs, 1× PCR buffer (Invitrogen), 3 mM MgCl₂, 2 µL diluted SYBR^®^ Green I (1:1000), and 0.25 U Platinum Taq DNA polymerase (Invitrogen). Cycling: 94 °C for 5 min; 40 cycles of 94 °C for 15 s, 60 °C for 10 s (with fluorescence acquisition), and 72 °C for 15 s. A melting-curve analysis followed. Housekeeping genes *UKN1* and *UKN2* were used for normalization. Primer specificity was confirmed by melting-curve analysis and agarose gel electrophoresis (data not shown). Primer efficiencies were calculated with Miner (Zhao and Fernald, 2005). Normalized relative quantities (NRQs) were computed with QBASE v1.3.5 (Hellemans et al., 2008). Fold-change values were clustered into a gene-expression similarity tree using Cluster 3.0 (de Hoon et al., 2004) with rank correlation and complete linkage; the tree was visualized in TreeView v.1.1.6.r4 (Saldanha, 2004).

### Generation of transgenic Arabidopsis plants overexpressing *GmBAM-like 1*

The Gateway strategy was used to clone the full gene sequence of *GmBAM-like 1* into the pENTR™/D-TOPO^®^ vector (Invitrogen) and, later, into the pK2GW7 vector. Initially, the full gene sequence of *GmBAM-like 1* was amplified from the cDNA in a reaction containing 0.5 μl of Phusion^®^ High-Fidelity DNA Polymerase, 10 μl of HF buffer (5x), 1 μl of dNTPs (10 mM), 0.2 µM of each primer (10 μM), 1 μl of cDNA and 32.5 μl of nuclease-free water. PCR reactions had the following cycling steps: initial denaturation at 98 °C for 30 s; followed by 30 amplification cycles of 98 °C for 10 s, 55 °C for 30 s and 72 °C for 1 minute; ending with a final 10 min extension at 72 °C. The PCR product was purified using the NucleoSpin^®^ PCR Clean-up kit (Macherey-Nagel). Cloning of *GmBAM-like 1* into the pENTR™/D-TOPO^®^ vector (Invitrogen) was performed using a molar ratio of 2:1 (insert:vector), following the manufacturer’s instruction. The integrity and orientation of the insert were confirmed by Sanger sequencing, using M13-pUC universal primers. Recombination into the pK2GW7 vector was performed using LR Clonase™ II (Invitrogen), using 150 ng of the pK2GW7 plasmid and 100 ng of the pENTR::*GmBAM-like 1* vector. Positive clones were identified by PCR with *GmBAM-like 1*-specific primers (Table S1). The pK2GW7::*GmBAM-like 1* construct, containing the *GmBAM-like 1* coding sequence under the Cauliflower Mosaic Virus 35S promoter, was introduced into *Arabidopsis thaliana* (Col-0) via *Agrobacterium tumefaciens*-mediated floral dip (Clough and Bent, 1998). Transformants were selected on Murashige and Skoog (MS) medium (Murashige and Skoog, 1962) containing kanamycin. T3 homozygous 35S::*GmBAM-like 1* lines were used for molecular and phenotypic analyses. Plants were grown in soil under controlled conditions (16/8 h light/dark; 100 µmol m⁻² s⁻¹; 22 °C ± 2 °C; 60% relative humidity).

### Evaluation of abiotic stress tolerance in transgenic Arabidopsis overexpressing *GmBAM-like 1*

T3 seeds of 35S::*GmBAM-like 1* lines and wild-type Col-0 were surface-sterilized and cold-stratified at 4 °C for four days prior to germination under controlled growth-chamber conditions (continuous photoperiod; 100 µmol m⁻² s⁻¹; 22 °C ± 2 °C; 60% RH). Salt and osmotic tolerance assays followed a modified protocol from Chung et al. (2013). Seeds were germinated on MS medium (0.8% agar) with or without 150 mM NaCl (salt stress) or 200 mM mannitol (osmotic stress). Germination rates were recorded on day 7. Three independent biological replicates were performed. Seedling survival under stress was evaluated by transferring 30 seven-day-old seedlings to fresh MS plates containing 200 mM NaCl or 200 mM mannitol; surviving seedlings were counted after five days (NaCl) or seven days (mannitol). Three independent replicates were conducted. Drought tolerance in vitro used PEG 8000 following Verslues and Juenger (2011): seeds germinated on half-strength MS agar were transferred to plates containing half-strength MS supplemented with 550 g L⁻¹ PEG 8000 and grown for an additional seven days. Malondialdehyde (MDA) content - an indicator of membrane damage - was measured as described by Hodges et al. (1999). Soil-based drought tolerance and recovery were assessed using a modified Van Houtte et al. (2013) protocol. Plants (wild-type Col-0 and transgenic lines AG2, D2, G6) were maintained near field capacity until three weeks old, after which watering was withheld. Visual wilting symptoms were monitored daily until ~90% of plants displayed severe symptoms; plants were then rewatered and recovery recorded. Three independent biological replicates were performed, each with 15 plants grown in individual pots. Leaf area was estimated using ImageJ (Schneider et al., 2012); fresh and dry weights of above-ground biomass were measured and tissue relative water content (RWC) calculated. Physiological parameters (transpiration rate and carbon assimilation) were measured with an infrared gas analyzer fitted with a custom Arabidopsis whole-plant cuvette. A photosynthetic photon flux density (PPFD) of 300 µmol m⁻² s⁻¹ was used for measurements. The measuring gas was calibrated to 20,000 ppm humidity and 400 ppm CO₂. Transpiration rate (E; mmol H₂O m⁻² s⁻¹) and photosynthetic rate (JCO₂; µmol CO₂ m⁻² s⁻¹) were normalized to the total leaf area of each sample.

### Quantification of starch in plant material

Starch was quantified from ethanol extracts using a method modified from Rovelet-Lecrux et al. (2006). Five biological replicates were analysed, each consisting of pooled shoots from six plants grown for 30 days under a 16 h light/8 h dark photoperiod. Ethanol-extraction pellets were resuspended in 0.1 M NaOH and heated at 95 °C for 30 min to gelatinize starch. The suspension was acidified to pH 6.0 with HCl/sodium acetate buffer (pH 4.9) and enzymatically digested overnight with amyloglucosidase and α-amylase to convert starch to glucose. Glucose in the supernatant was quantified to estimate total starch.

### Statistical analysis

Multivariate analyses were performed in R to interpret RT-qPCR gene-expression profiles and included hierarchical clustering heatmaps, PCA, and Random Forest. Fold-change values were calculated as treatment/control ratios and log₁₀-transformed for normalization; these normalized values were used for downstream analyses. To assess overall stress response, data from distinct stress treatments within each cultivation system (PSys and HSys) were combined into a single “water stress” group; statistical comparisons between different stress levels were not performed. Morphological and physiological data are presented as mean ± standard error (SE). Pairwise comparisons used Student’s t-test; multiple comparisons were analyzed by ANOVA followed by Tukey’s post-hoc test. A p-value ≤ 0.05 was considered statistically significant.

## Results

### Comprehensive analysis of drought tolerance mechanisms in soybean: gene expression profiling in roots and leaves

Our study began with an extensive *in silico* analysis that identified 80 drought-responsive genes in *Arabidopsis*, which belonged to 39 different metabolic pathways. By applying stringent criteria to find soybean homologs, we obtained a pool of 354 putative soybean orthologous genes potentially involved in the drought response (Guimarães-Dias et al., 2012). The expression pattern of the 354 genes was evaluated using Solexa cDNA subtractive libraries from multiple soybean cultivars exposed to various water deficit treatments (Rodrigues et al., 2012). By selecting the genes based on their presence in the libraries related to drought stress, we narrowed the genes to a final set of 21 high-confidence drought-tolerance candidate genes (Figure S2). We then performed detailed expression profiling of these genes by RT-qPCR. This integrative pipeline provides strong confidence in the selection of drought-responsive targets and establishes a solid basis for functional follow-up.

**Figure 1 -:**
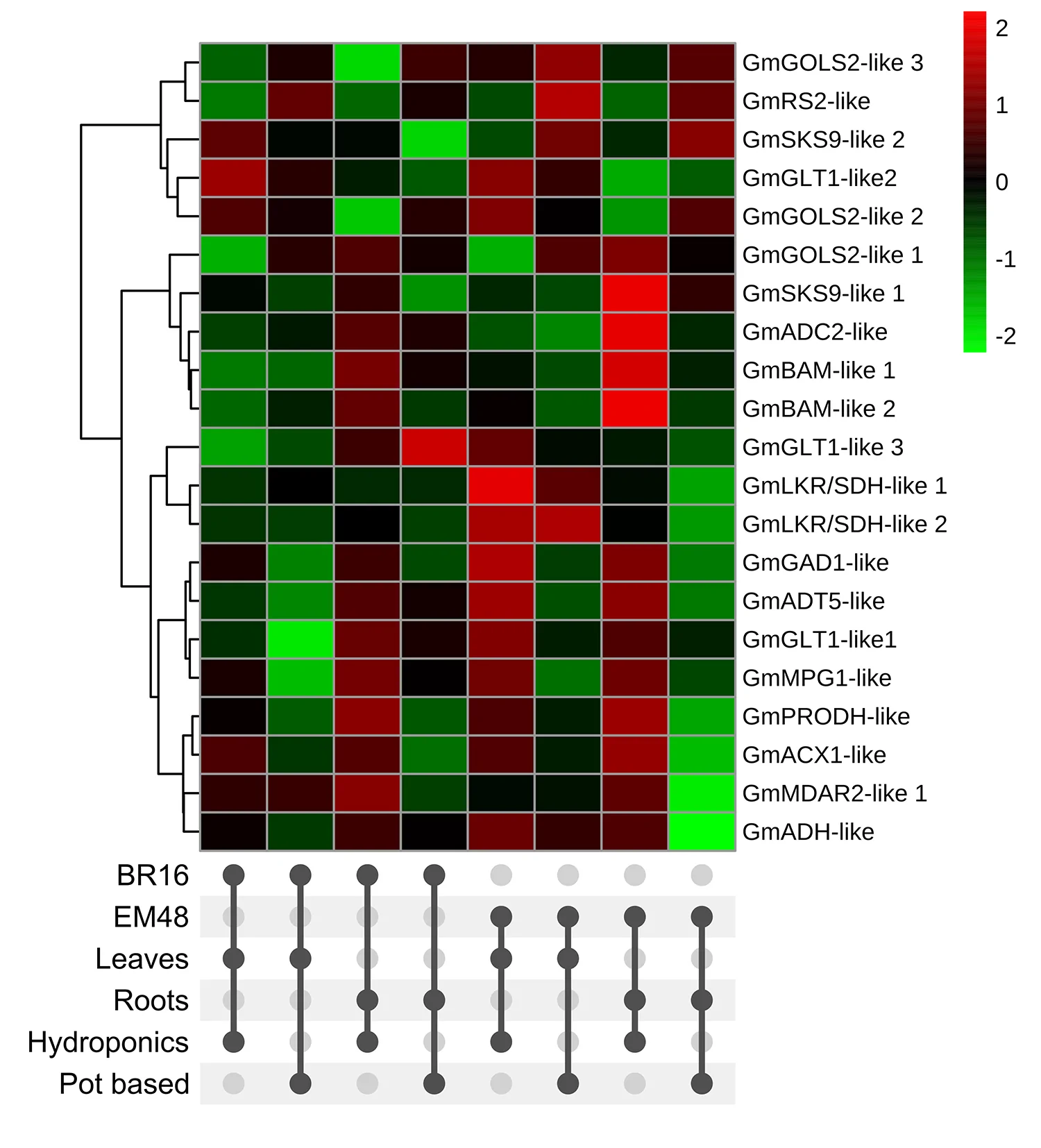
Hierarchical clustering heatmap of gene expression under drought stress. Heatmap showing log₁₀-transformed fold-change (treatment/control) of the 21 candidate drought-responsive genes across two soybean cultivars (Embrapa 48 = tolerant; BR16 = sensitive), two tissues (leaves, roots) and two cultivation systems (pot-based, PSys; hydroponic, HSys). Normalized relative quantities (NRQs) from RT-qPCR were used as input and fold-changes were log₁₀-transformed prior to clustering. Genes (rows) and samples (columns) were clustered using rank-correlation and complete-linkage clustering in Cluster 3.0; the tree was visualized with TreeView. Color scale indicates log₁₀(fold-change) (green = downregulated; black = no change; red = upregulated). Two independent biological replicates per condition were used; each RT-qPCR reaction was run in technical triplicate (see Methods). Statistical comparisons and normalization procedures are described in Materials and Methods.

**Figure 2 -:**
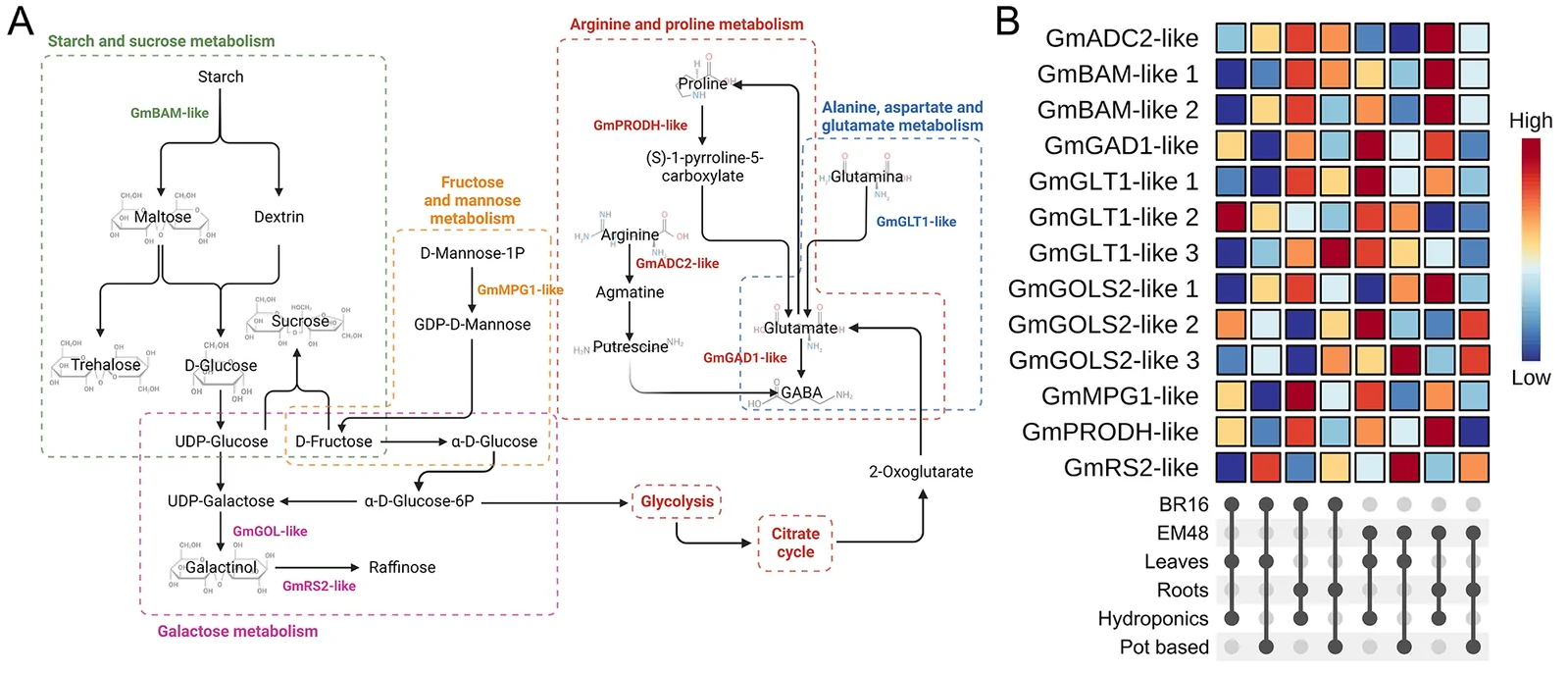
Metabolic mapping and expression profiles of differentially expressed genes. A. Schematic metabolic map showing the position of the differentially expressed genes within carbohydrate metabolism (starch and sucrose; fructose and mannose; galactose metabolism) and the GABA shunt and related pathways (arginine and proline metabolism; alanine, aspartate, and glutamate metabolism). Pathway annotation was based on AraCyc and KEGG; genes identified in this study are highlighted and colored by pathway. B. RT-qPCR expression profiles (fold change: treatment / control) of up-and down-regulated genes in leaves and roots of Embrapa 48 and BR16 grown in PSys and HSys. Bars represent mean ± SE (n = 2 biological replicates; each biological sample comprises technical triplicates). Color scale indicates log₁₀(fold-change) (blue = downregulated; white = no change; red = upregulated). Fold-changes were calculated from NRQs (QBASE) and log-transformed for visualization. Statistical tests used are indicated in Materials and Methods; significant differences between cultivar/treatment combinations were determined as described in Material and Methods. Image created in https://BioRender.com.

Of the 21 genes identified (Figure 1), seven are closely linked to carbohydrate metabolism (*GmGOLS2-like 1*, *GmGOLS2-like 2*, *GmGOLS2-like 3*, *GmRS2-like*, *GmBAM-like 1*, *GmBAM-like 2*, and *GmMPG1-like*), while six belong to the GABA shunt pathway (*GmGLT1-like 1*, *GmGLT1-like 2*, *GmGLT1-like 3*, *GmADC2-like*, *GmGAD1-like*, and *GmPRODH-like*) (Figure 2 A). Because these two functional categories account for 61.9% of the candidates, we focused subsequent analyses on them, underscoring their likely central role in soybean drought responses. Their respective gene codes are listed in Table S2.

Expression profiling of these 13 genes revealed distinct tissue- and system-specific patterns. Under hydroponic conditions (HSys), five genes (*GmADC2-like*, *GmBAM-like 1* and *2*, *GmGOLS2-like 1*, and *GmPRODH-like*) were upregulated in roots of the tolerant cultivar Embrapa 48. In contrast, *GmGAD1-like*, *GmGLT1-like 1*, and *GmGOLS2-like 2* showed peak expression in Embrapa 48 leaves under HSys. Under pot-based conditions (PSys), *GmGOLS2-like 3* and *GmRS2-like* were most highly expressed in Embrapa 48 roots. In the sensitive cultivar BR16, fewer genes were HSys-responsive: *GmGLT1-like 2* was higher in leaves, and *GmMPG1-like* was higher in roots; *GmGLT1-like 3* peaked in BR16 roots under PSys (Figure 2 B ). Among the ten genes most strongly induced in HSys (*GmGLT1-like 2, GmMPG1-like, GmGAD1-like, GmGLT1-like 1, GmGOLS2-like 2, GmADC2-like, GmBAM-like 1, GmBAM-like 2, GmGOLS2-like 1,* and *GmPRODH-like*), eight showed higher expression in Embrapa 48 versus only two in BR16. These results are consistent with the higher tolerance to water deficit in Embrapa 48 and indicate that, in our experimental conditions, the water deficit induction through HSys was effective at revealing putative drought response genes, that could be further validated.

### Comparative analysis of drought stress responses: insights from drought-tolerant and sensitive soybean lineages

**Figure 3 -:**
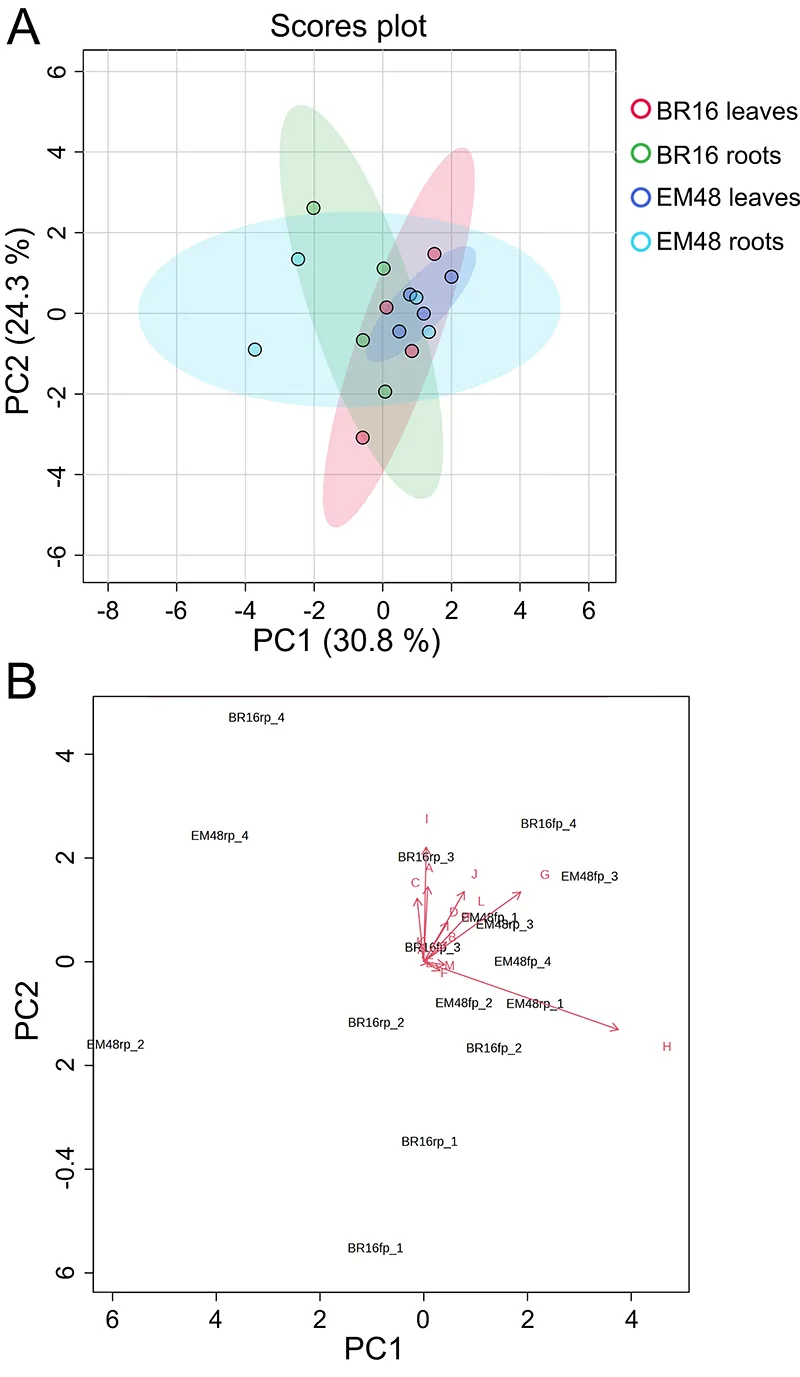
Principal Component Analysis (PCA) of samples from the Pot-Based System (PSys). A. Two-dimensional score plot (PC1 vs PC2) showing sample clustering by cultivar (Embrapa 48, BR16) and tissue (leaf, root) under PSys drought treatments. Input data were log₁₀-transformed fold-change NRQs from RT-qPCR. Percent variance explained by PC1 and PC2 is shown on the axes. B. PCA biplot (loadings) illustrating the contribution of individual genes to sample separation; arrows indicate gene loadings and their direction/length reflects the magnitude of influence on sample clustering. Gene labels: A = *GmGOLS2-like 3*; B = *GmADC2-like*; C = *GmBAM-like 1*; D = *GmGLT1-like 1*; E = *GmBAM-like 2*; F = *GmGAD1-like*; G = *GmPRODH-like*; H = *GmGLT1-like 2*; I = *GmGOLS2-like 2*; J = *GmGOLS2-like 1*; K = *GmMPG1-like*; L = *GmRS2-like*; M = *GmGLT1-like 3*. PCA was computed in R (see Methods); input normalization and transformation are described in Materials and Methods. Principal Component (PC) Loadings are available in Table S3.

**Figure 4 -:**
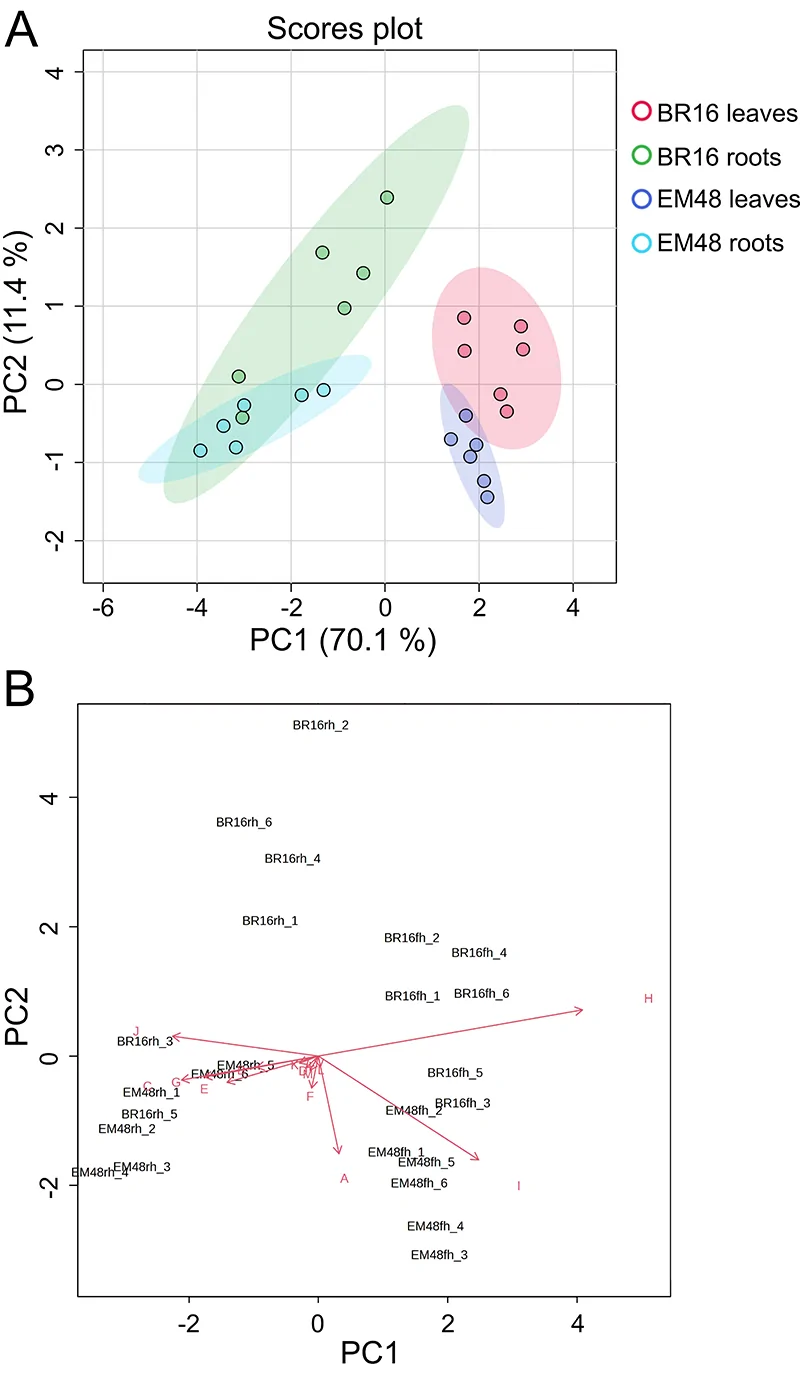
Principal Component Analysis (PCA) of the Hydroponics-Based System (HSys). A. Two-dimensional PCA score plot (PC1 vs PC2) showing clustering of samples from Embrapa 48 and BR16 (leaf and root) under HSys drought treatments. Percent variance explained by PC1 and PC2 is shown on the axes. B. PCA biplot showing gene loadings and their influence on sample separation. Note that *GmGOLS2-like 2* and *3* drive leaf clustering in Embrapa 48, while *GmBAM-like 1/2* and *GmPRODH-like* contribute strongly to root clustering (see Results). A = *GmGOLS2-like 3*; B = *GmADC2-like*; C = *GmBAM-like 1*; D = *GmGLT1-like 1*; E = *GmBAM-like 2*; F = *GmGAD1-like*; G = *GmPRODH-like*; H = *GmGLT1-like 2*; I = *GmGOLS2-like 2*; J = *GmGOLS2-like 1*; K = *GmMPG1-like*; L = *GmRS2-like*; M = *GmGLT1-like 3*. Methods for PCA and data preprocessing are given in Materials and Methods. Principal Component (PC) Loadings are available in Table S4.

To characterize global expression patterns, we applied Principal Component Analysis (PCA) to genes in carbohydrate metabolism and the GABA shunt pathway. PCA revealed distinct lineage responses that were corroborated by Random Forest Analysis (RFA) (Figure S3). In the PSys (Figures 3 A and 3B), treatments largely overlapped, indicating similar responses among the analyzed genes. By contrast, HSys (Figure 4 A) produced a clear separation between Embrapa 48 and BR16, particularly in leaf tissue, indicating divergent responses to rapid water deprivation in hydroponics. We also observed marked differences between leaf and root expression within each cultivar.

In Embrapa 48 (Figure 4 B ), *GmGOLS2-like 2* and *3* were major contributors to leaf clustering, whereas *GmBAM-like 1* and *2* and *GmPRODH-like* drove root clustering. In BR16 (Figure 4 B ), *GmGLT1-like 2* was the principal variable contributing to leaf clustering. RFA (Figure S3) confirmed these contrasting responses in HSys: 11 of 13 genes were more highly expressed in Embrapa 48. Of these, six (*GmGOLS2-like 3*, *GmGOLS2-like 2*, *GmGLT1-like 1*, *GmGAD1-like*, *GmRS2-like*, and *GmGLT1-like 3*) were predominantly expressed in leaves, while five (*GmGOLS2-like 1*, *GmPRODH-like*, *GmBAM-like 2*, *GmBAM-like 1*, and *GmADC2-like*) were more abundant in roots. Importantly, six of the seven top-ranking genes in both varieties were implicated in carbohydrate metabolism, underscoring the central role of carbohydrate pathways in drought tolerance (Figure S3).

### Identification of root-specific drought response genes in soybean

Early root responses are particularly informative because roots are the primary sensors of soil water deficit (Vadez, 2014). Expression profiles (Figure 1) identified *GmBAM-like 1* and *2* as strongly upregulated in HSys root tissue, ranking among the highest expressed genes of the 21 candidates. Both genes heavily influenced the clustering of HSys root samples from the tolerant cultivar (Figure 4 B ) and were identified as key drought-responsive variables by RFA (Figure S3). Their similar expression patterns across treatments (Figure 1) indicate that *GmBAM-like 1* (hereafter *GmBAM-like 1.1*) and *GmBAM-like 2 (GmBAM-like 1.2*) likely perform comparable functions under water limitation. Zanella et al. (2016) suggest that carbon skeletons released by transient diurnal starch degradation via *AtBAM1 (At3g23920)* support proline biosynthesis required for osmotic adjustment; *atBAM1* has also been implicated in mesophyll starch turnover during drought or salt stress (Valerio et al., 2011; Monroe and Storm, 2018).

**Figure 5 -:**
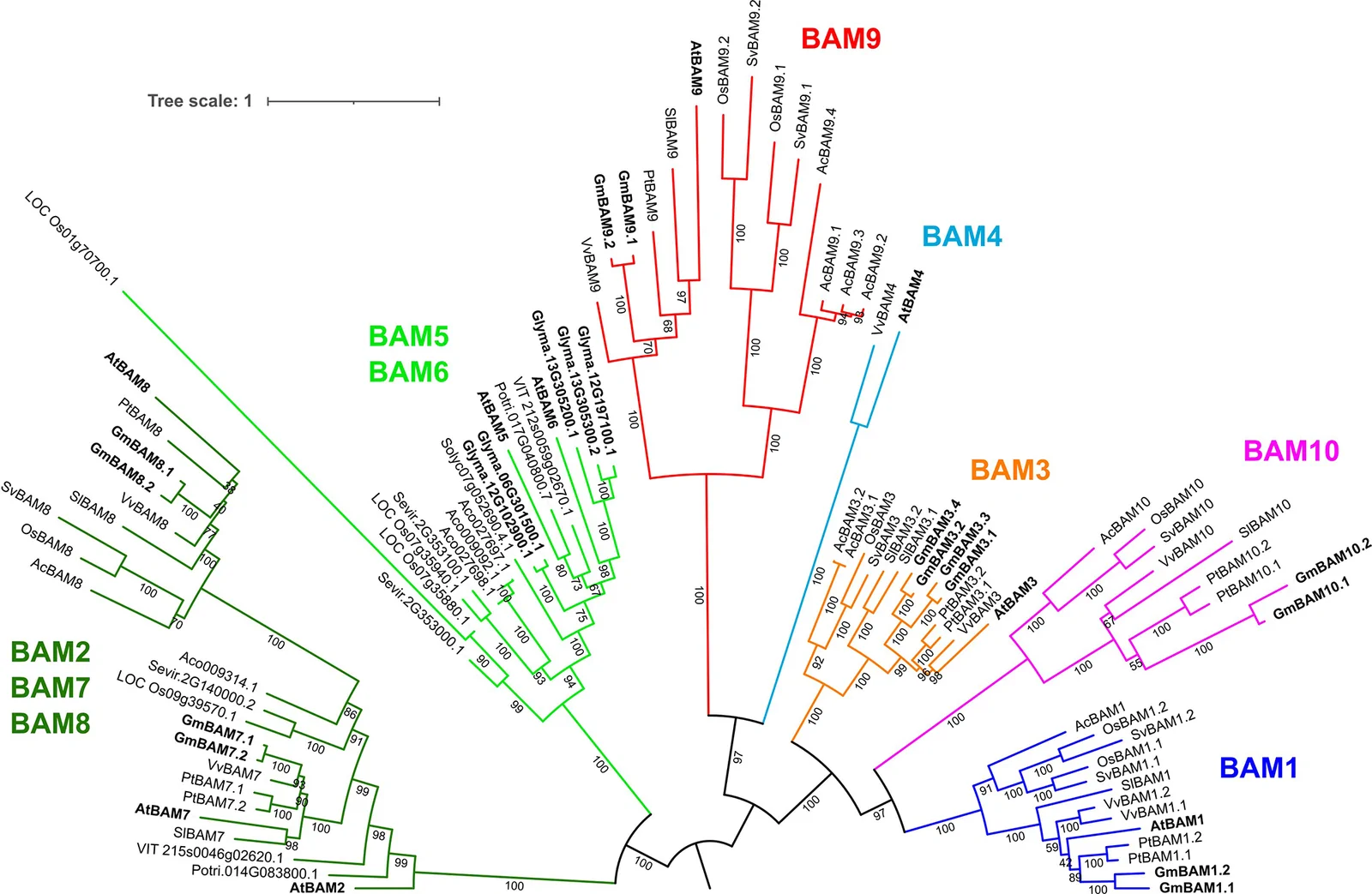
Maximum-likelihood phylogenetic tree of β-amylase protein sequences. Maximum-likelihood tree inferred using IQ-TREE with the JTT+F+R7 substitution model (ModelFinder) and bootstrap support of 2,000 replicates. Full-length β-amylase sequences from *Ananas comosus* (Ac), *Arabidopsis thaliana* (At), *Glycine max* (Gm), *Oryza sativa* (Os), *Populus trichocarpa* (Pt), *Setaria viridis* (Sv), *Solanum lycopersicum* (Sl), and *Vitis vinifera* (Vv) were aligned with MUSCLE prior to phylogenetic inference. Seven major clades are indicated by color; proteins from *A. thaliana* and *G. max* are highlighted in bold. Nodes with bootstrap support < 70% are marked with an asterisk. The tree was midpoint rooted. Correspondence between protein labels and Phytozome identifiers is provided in Table S5. Methods and model selection details are described in Material and Methods.

To confirm the homology, we constructed a maximum-likelihood tree of β-amylases sequences. Protein sequences from *A. comosus, A. thaliana, G. max, O. sativa, P. trichocarpa, S. viridis, S. lycopersicum,* and *V. vinifera* were aligned. The tree recovered seven well-supported clades corresponding to known *A. thaliana* BAM groups (except BAM10, which lacks an Arabidopsis homolog) (Figure 5). The overall topology of the tree was similar to other trees from previous studies (Thalmann et al., 2019). All clades included genes from monocot and dicot species, with the exception of the BAM4 clade (Figure 5), which is absent in monocotyledon species. It is worth noting that the clades had distinct branch lengths, which indicates different degrees of conservation among the genes of each clade. The clade corresponding to BAM1 had short branch lengths, for example, indicating that BAM1 orthologues from different species are highly conserved (Figure 5). Soybean has two BAM1 genes (*GmBAM-like 1.1* and *GmBAM-like 1.2*), both of which clustered as orthologues of *AtBAM1*. Since *GmBAM-like 1.1* showed the highest sequence similarity to *AtBAM1* in Phytozome, it was selected for heterologous overexpression in *Arabidopsis thaliana* to evaluate its potential for improving drought tolerance (Figure S2).

### The protective role of *GmBAM-like 1*: Enhancing stress tolerance and reducing oxidative damage in transgenic Arabidopsis

To assess function, we generated transgenic Arabidopsis lines expressing 35S::*GmBAM-like 1*. PCR verified transgene insertion, and three independent T3 lines (AG2, D2, G6) were analyzed further (Figure S4). Under control conditions, transgenics and wild-type (WT) plants showed no notable morphological differences (silique morphology, length, or seed weight) (Figure S4). Given the overlap among salinity, osmotic, and drought response pathways, we evaluated the transgenics under these stresses.

**Figure 6 -:**
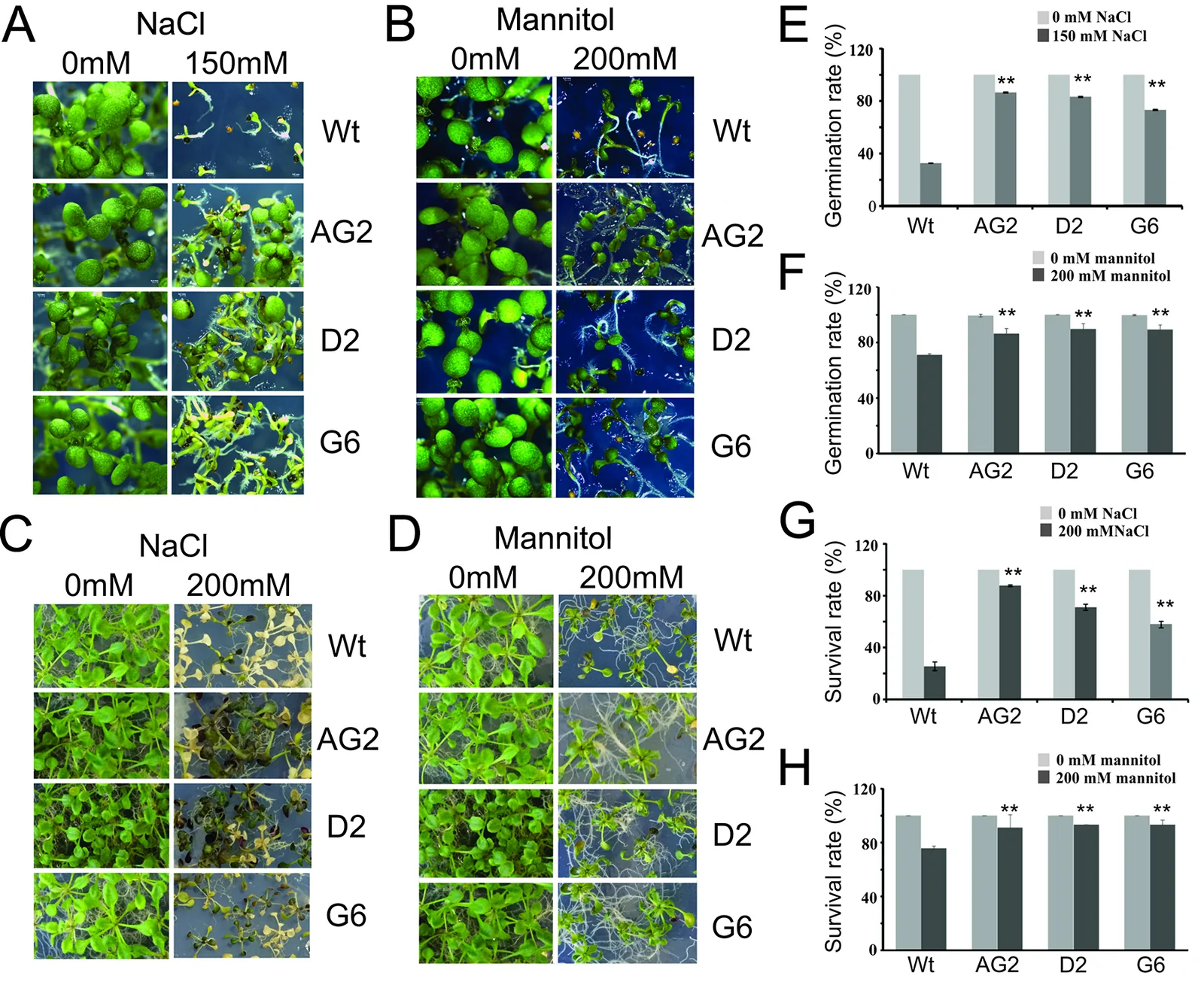
Germination and survival of Arabidopsis lines overexpressing *GmBAM-like 1* under saline and osmotic stress**.** A, B, E and F. Germination rates (%) on MS medium supplemented with 150 mM NaCl (A and E) or 200 mM mannitol (B and F). Data represent mean ± SE from three independent biological replicates; each replicate comprised n = 30 seeds per genotype (total n indicated on each bar). C, D, G and H. Seedling survival (%) following transfer to MS medium containing 200 mM NaCl (C and G) or 200 mM mannitol (D and H). For survival assays, n = 100 seeds were evaluated per genotype and results are presented as mean ± SE of three biological replicates (see Methods). Statistical significance (Student’s t-test) comparing each transgenic line to wild type (WT) is indicated by ** (p < 0.05). See Methods for germination and survival scoring criteria.

*GmBAM-like 1* overexpression did not impair growth and produced substantial stress benefits. On MS medium containing 150 mM NaCl, germination of transgenic seeds ranged from 73% to 87%, compared with ~33% for WT (Figure 6 A and 6E ). Transgenics also germinated better on 200 mM mannitol (Figure 6 B and 6F ). Survival after stress likewise increased: transgenic survival ranged from 58% to 88% on 200 mM NaCl versus 26% for WT (Figure 6 C and 6G ); similar improvements were observed after 7 days on mannitol, although symptoms were milder (Figure 6 D and 6H ).

**Figure 7 -:**
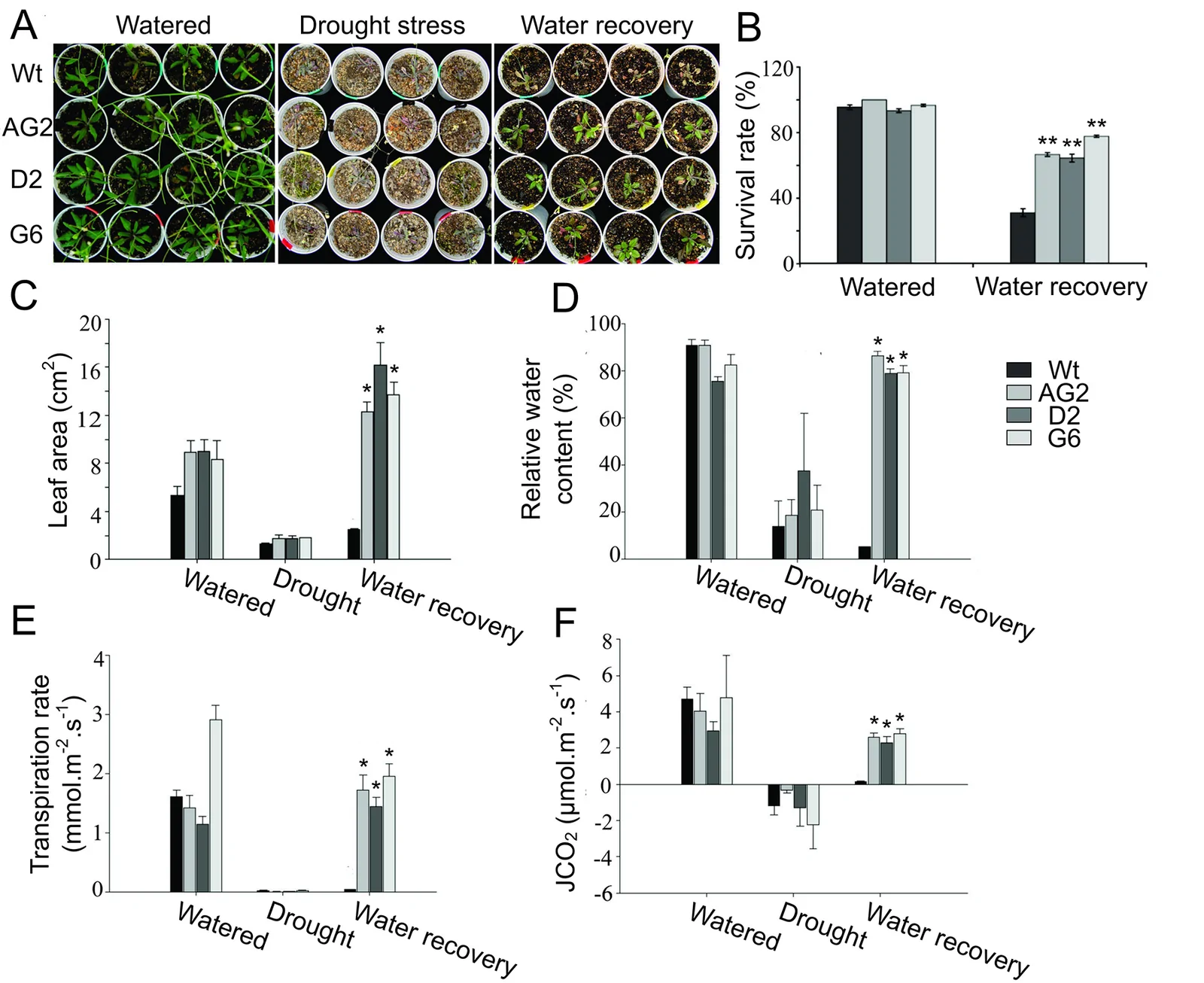
Drought response and recovery of *GmBAM-like 1* transgenic Arabidopsis. A. Representative photographs of WT and transgenic lines (AG2, D2, G6) under well-watered conditions, after drought treatment, and 48 h after rewatering (recovery). Representative images correspond to plants used in physiological assays. B. Survival rate 48 h after rewatering. Data are means ± SE from three biological replicates (n = 15 plants per replicate). Statistical comparison between WT and transgenics was performed by Student’s t-test; significance symbols: * p < 0.05; + p < 0.10. C. Total leaf area (cm²) measured by ImageJ for plants under control, drought and recovery (n = 8 per genotype; mean ± SE). D. Relative water content (RWC, % of water content per dry mass) under the three conditions (n = 8; mean ± SE). E. Transpiration rate (mmol H₂O m⁻² s⁻¹) normalized to total leaf area (n = 8; mean ± SE). F. Net photosynthetic rate (µmol CO₂ m⁻² s⁻¹) measured under standardized conditions (PPFD = 300 µmol m⁻² s⁻¹) (n = 8; mean ± SE). Morphological and physiological data were analyzed by one-way ANOVA followed by Tukey’s post-hoc test; different letters (or symbols as indicated) denote statistically significant differences at α = 0.05. Survival data were compared by Student’s t-test (WT vs each transgenic line).

**Figure 8 -:**
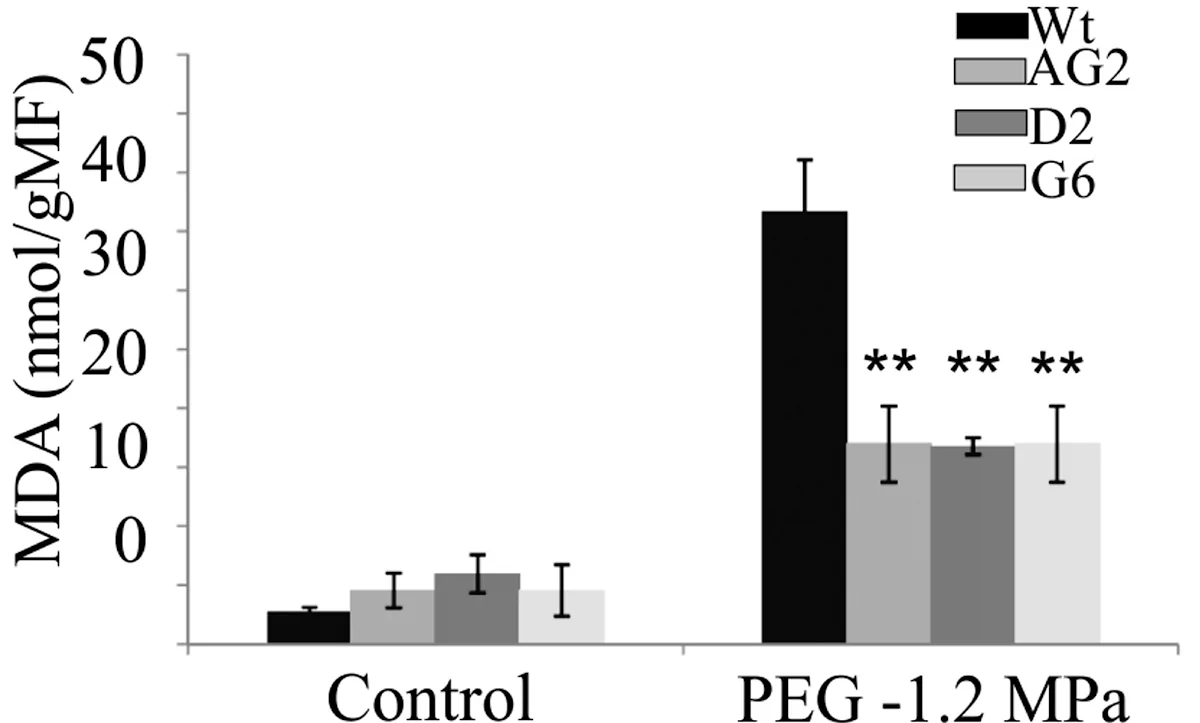
Malondialdehyde (MDA) accumulation in WT and *GmBAM-like 1* transgenic plants after PEG-induced water deficit. Leaf MDA content (mg MDA g⁻¹ FW) measured after exposure to −1.2 MPa PEG 8000 treatment (see Methods). Values are means ± SE of three biological replicates (n = 15 plants total per genotype). MDA was quantified according to Hodges et al. (1999). A Student’s t-test comparing WT and each transgenic line is shown; ** denotes p < 0.05. Reduced MDA in transgenics indicates lower lipid peroxidation and oxidative damage under water deficit.

Drought tolerance assays (soil and PEG-supplemented plates) revealed that both genotypes experienced comparable stress severity (leaf purpling, reduced transpiration, >60% loss of above-ground water content, ~75% leaf area reduction, and impaired carbon assimilation) (Figure 7 C-F ). However, following rewatering, transgenic plants recovered more robustly: survival ranged from 66% to 77% versus 31% in WT (Figure 7 A-B ). Transgenic plants restored relative water content to >80% (Figure 7D) and regained pre-stress transpiration rates (Figure 7 E ). Photosynthetic recovery showed a trend favoring transgenics (p = 0.07) (Figure 7 F). Importantly, leaf malondialdehyde (MDA) accumulation-a marker of oxidative damage-was substantially lower in transgenics (18 mg g⁻¹ FW) than in WT (37 mg g⁻¹ FW) (Figure 8), indicating reduced lipid peroxidation after stress.

Starch assays showed no difference in end-of-day (ED) synthesis, but at end-of-night (EN) transgenic lines generally had lower starch levels than WT (except line G6). Nevertheless, transgenics exhibited higher starch degradation rates than WT (Table 1), consistent with enhanced BAM activity and a mechanism that likely contributes to improved osmotic adjustment and stress recovery.

**Table 1 -:** Starch levels and percentage of degradation in BAM1 overexpressing transgenic lineages and wild-type Arabidopsis. Starch levels and the degradation percentage were assessed in transgenic lineages overexpressing BAM1 and the wild-type counterparts of Arabidopsis. The average starch levels for each period were calculated using data obtained from five biological replicates (n=6). Statistical analysis was conducted using the Student’s t-test (α < 0.05) to determine significant differences (*) in starch levels. ED = end of day; EN = end of night.

Genotype	Time of the Day		
	ED	EN	
	µmol/gFW		% of degradation
WT	18.6 ± 3.2	5.9 ± 0.6	68.3
AG2	18.1 ± 1.3	1.0 ± 0.2*	94.4
D2	23.2 ± 1.7	3.8 ± 0.4*	83.6
G6	28.38 ± 4.43	7.7 ± 0.69	72.8

## Discussion

In this study, we examined the expression of genes from several metabolic pathways, with particular emphasis on those related to carbohydrate metabolism and the GABA shunt, which together accounted for more than 60% of the genes analyzed. Experiments were conducted in both pot-based (PSys) and hydroponic (HSys) systems using the drought-tolerant cultivar Embrapa 48 and the drought-sensitive cultivar BR16. Using two contrasting water-deprivation systems proved informative, revealing different facets of the plant drought response. While the PSys is more similar to field conditions, since there is a gradual depletion of the water from the soil, the water deficit in the HSys by air-drying imposes a rapid water-deprivation stimulus. These observations have important implications for breeding drought-tolerant soybean varieties (Guimarães-Dias et al., 2012; Neves-Borges et al., 2012; Tripathi et al., 2015). Consistent with these system-dependent dynamics, gene expression patterns differed markedly between cultivation methods (Figures 3-4). In the PSys, both cultivars displayed broadly similar expression profiles for carbohydrate- and GABA-related genes (Figure 3). Under HSys conditions, however, the cultivars diverged (Figure 4). The tolerant cultivar Embrapa 48 showed notably higher expression for 11 genes compared with the susceptible variety (Figure S3). These results indicate that Embrapa 48 employs a proactive strategy, rapidly activating carbohydrate-metabolism and GABA-shunt genes in anticipation of water deficit. Such early activation likely contributes to the enhanced drought tolerance observed in this cultivar.

BAMs catalyze starch breakdown and are central to plant energy and carbon remobilization under abiotic stress. Starch turnover is strongly affected by drought, osmotic stress, and temperature fluctuations (Peng et al., 2014; Yang et al., 2023; Zhang Y. et al., 2023). When photosynthesis is compromised, plants remobilize starch to maintain a supply of energy and carbon for essential metabolism (Zeeman et al., 2010; Dong and Beckles, 2019; Ďúranová et al., 2023). BAM-mediated starch degradation releases sugars and derived metabolites that support growth under stress and act as osmoprotectants and compatible solutes (Zanella et al., 2016; Thalmann and Santelia, 2017; S. Dong and Beckles, 2019).

The *A. thaliana* genome encodes nine BAM isoforms (BAM1-9) grouped into seven clades (Thalmann et al., 2019). Other species additionally possess a BAM10 clade, which is absent in *A. thaliana* and other Brassicales. Our maximum-likelihood analysis of BAM proteins from multiple species (Figure 5) produced a topology consistent with Thalmann *et al*. (2019). The soybean genome encodes all BAM isoforms, with the exception of BAM4, which is absent in Fabaceae and monocot species. Since BAM4 plays a key role in starch degradation (Fulton et al., 2008), these species probably have alternative pathways of starch degradation. Among the recovered clades, BAM1 and BAM3 exhibit a high degree of conservation among the genes, indicating that these two BAMs may be under stronger purifying selection, when compared with the other isoforms. Six isoforms (BAM1-4, BAM6, and BAM10) are predicted to be chloroplast localized, though not all directly mediate starch degradation (Thalmann *et al*., 2019). Importantly, BAM1 has been reported as a major contributor to diurnal starch breakdown and to plant responses to drought, salinity, and osmotic stress (Kaplan and Guy, 2004; Valerio et al., 2011; Zanella et al., 2016; Zi et al., 2022), highlighting the functional diversity of BAM isoforms and supporting a specific role for *GmBAM-like 1* in stress tolerance.

Our functional analysis of *GmBAM-like 1* provides compelling evidence of its contribution to stress tolerance. Transgenic Arabidopsis lines overexpressing the soybean *GmBAM-like 1* exhibited higher germination and survival rates under salt (NaCl) and osmotic (mannitol) stress than wild type (Figure 6). This improvement is consistent with *GmBAM-like 1*-mediated starch hydrolysis producing maltose, a precursor for osmoprotectant synthesis (e.g., galactinol and raffinose), which accumulates in transgenic plants and enhances stress tolerance (Nishizawa-Yokoi et al., 2008; Zúñiga-González et al., 2016; Dong and Beckles, 2019). Increased maltose likely facilitated osmotic adjustment and helped mitigate oxidative damage, thereby improving germination and early survival. These findings align with reports that *AtBAM1* supports light-induced starch degradation and provides carbon skeletons for sucrose and proline biosynthesis during stress (Zanella et al., 2016; Lemonnier and Lawson, 2024).

Overexpression of *GmBAM-like 1* also improved drought recovery. Under water limitation, both transgenic and wild-type plants showed reductions in leaf area, relative water content (RWC), photosynthetic rate, and transpiration (Figures 7 C-F ). However, following rehydration transgenic lines recovered more rapidly and completely: they displayed higher survival (66-77% vs. 31% in WT; Figures 7 A-B), regained RWC to >80% (Figure 7 D), and restored pre-stress transpiration rates (Figure 7 E ). Photosynthetic recovery also trended higher in transgenics (p = 0.07; Figure 7 F ). These improvements are attributable, at least in part, to enhanced starch turnover in *GmBAM-like 1* lines that increases maltose and glucose availability and may promote stomatal opening after rehydration.

Stomatal conductance is governed by guard-cell turgor, which is influenced by ion fluxes and osmolyte concentrations (H. Dong et al., 2018). Stress-activated anion channels (e.g., SLAC1) and subsequent K⁺ efflux contribute to stomatal closure (Hedrich, 2012; Roelfsema et al., 2012). Interconversion between starch and malate in guard cells is also central to stomatal dynamics: conversion of malate to starch supports closure, whereas starch degradation to glucose is an early step in stomatal opening (Daszkowska-Golec and Szarejko, 2013; H. Dong *et al*., 2018; Flütsch et al., 2020). The reduced end-of-night starch levels observed in transgenic lines (AG2 and D2; Table 1), likely a result of *GmBAM-like 1* activity, would increase soluble carbohydrates (glucose and maltose) and favor stomatal opening, thereby improving post-drought recovery. This hypothesis is supported by studies showing that *AtBAM1* mutants accumulate guard-cell starch and have impaired stomatal opening, underscoring the role of thioredoxin-regulated BAM1 in diurnal starch breakdown and stomatal function (Valerio et al., 2011; Horrer et al., 2016). 

Moreover, transgenic plants accumulated significantly less malondialdehyde (MDA) than wild type under PEG-induced water deficit (Figure 8), suggesting that *GmBAM-like 1* overexpression indirectly reduces oxidative damage. The likely mechanism is enhanced production of osmoprotectants via accelerated starch degradation, which can buffer cells against oxidative stress induced by drought and salinity (Nishizawa-Yokoi et al., 2008; Zúñiga-González et al., 2016; Dong and Beckles, 2019). Thus, *GmBAM-like 1*’s contribution to drought tolerance is multifaceted, combining improved stomatal regulation with reduced oxidative stress.

In summary, our results support a central role for carbohydrate metabolism and the GABA shunt in soybean drought responses. Embrapa 48 exhibits an accelerated stress response through rapid gene activation, with *GmBAM-like 1* emerging as a prominent drought-responsive gene, especially in roots under hydroponic stress. Heterologous overexpression of *GmBAM-like 1* in Arabidopsis conferred improved tolerance to drought, salinity, and osmotic stress, evidenced by higher germination and survival and enhanced recovery after rehydration. Though the functional validation has been limited to Arabidopsis, our initial results suggest that *GmBAM-like 1* has potential for improving tolerance to water deficit, salt, and osmotic stress. In the future, further studies in other plant species are needed to validate *GmBAM-like 1* as a promising candidate for inclusion in breeding programs aimed at improving tolerance to water deficit in crop plants.

## Supplementary material

The following online material is available for this article:

Table S1 - Primer sequences.

Table S2 - Gene codes for the soybean genes.

Table S3 - Principal Component (PC) Loadings of the Pot-Based System (PSys).

Table S4 - Principal Component (PC) Loadings of the Hydroponics-Based System (HSys).

Table S5 - Genes used in phylogenetic analysis, with species specifications and codes.

Figure S1 - Soybean plants growing in the hydroponic system.

Figure S2 - Gene selection strategy.

Figure S3 - Random Forest analysis of gene importance in the hydroponic system (HSys).

Figure S4 - Confirmation of transgene expression and morphological comparison between *GmBAM-like 1*-overexpressing lines and WT.

## Data Availability

 The entire dataset supporting the results of this study was published in the article itself.
