# Supplementary material for: Root-enriched β-amylase GmBAM-like 1 enhances drought and salt tolerance in Arabidopsis
Source: Genet Mol Biol. 2026 Aug 14;49(Suppl 3):e20250234. doi: 10.1590/1678-4685-GMB-2025-0234 (PMC13544623; doi:10.1590/1678-4685-GMB-2025-0234)
Supplement: Supplementary file 1 [file 1415-4757-GMB-49-s3-e20250234-s1.pdf]

Genetics and Molecular Biology
https://doi.org/10.1590/1678-4685-GMB-2025-0234

Supplementary Material to "Root-enriched β-amylase *GmBAM1* enhances drought and salt tolerance in Arabidopsis"

Table S1 - Primer sequences.

Gene code	Abbreviated code	Forward primer (5' – 3')	Reverse primer (5' – 3')
Glyma.03G137900	GMRS2-like	TGCTGGATGGTGCAAGATAG	CCTTTTGGTAGCCGAATCAC
Glyma.11G097300	GmSKS9-like 1	TGGTGTGTGGACGCCTAATA	ATTGCGGTCCATGACTTAGG
Glyma.06G019800	GmSKS9-like 2	GCAATTCCCAGGTCCAGATA	CCATCTTCGAACGAGTTCCT
Glyma.04G236900	GmGLT1-like 1	GCTGTAGGAGCCACAAAACC	GTGTCACCCCCACCAATAAC
Glyma.14G162300	GmGLT1-like 2	ATTGAGGGATCGGAGGAGAT	CACACCCAACTTCTCTGCAA
Glyma.19G065600	GmGLT1-like 3	ATTTCAGAGGGACGACAAGC	AAATCAGAGGAGCCACAAGG
Glyma.20G094500	GmGOLS2-like 1	CCTGAGAACGTTGAGCTTGA	CCACCACTTCTTCACCAACA
Glyma.03G222000	GmGOLS2-like 2	AGTCACCACTCCCACTTCGT	CCCGTATATCTCCACGGTTT
Glyma.19G219100	GmGOLS2-like3	TTGCCATGGCTTATTACGTC	TACCTCAATGTCTCCGTCCA
Glyma.06G007500	GmADC2-like	CAGGAGTATGTCAGCCACGA	CAGATCTTGAGCAGCAGGAA
Glyma.09G168300	GmBAM-like 1	GACGATCCCCAGTTCAATGT	TCTGGGTACGAAGGGTAACG
Glyma.16G217900	GmBAM-like 2	GTGAGCTGCGTTACCCTTCT	CATTCAGGCTTACCCTCAGC
Glyma.13G115500	GmLKR/SDH-like 1	GGGGAATGGTGTGATATGCT	ATTGGCTATGCAAGCTCTCC
Glyma.17G044300	GmLKR/SDH-like 2	ATCCTGCCACCTACAAATGG	ACGGAAAATGGTTGATGCTT
Glyma.18G043600	GmGAD1-like	CCACTCAACCTTCGCTTCTC	TATGCTGCCTCCTTCGGTAT
Glyma.13G319000	GmADT5-like	CACACGACAAAGGAACTTCG	CTAACCTCAGCCATGGAAGC
Glyma.02G250800	GmMPG1-like	CCTGGCTCTTGCTAGGGATA	CCTCTCCTCCATGGCTTTTA
Glyma.19G043000	GmPRODH-like	CCCGGTTCAATTTCAGTTGT	ACTTGAAACCCTGCATTGCT
Glyma.11G035200	GmACX1-like	CTGTTTCTCAGCTGGGCTCT	TCAACCAATCCTCAGCCTTT
Glyma.08G017800	GmMDAR2-like 1	TGCTGTGACTCCCTTTGAGA	CATGATCCACATGCTCCACT
Glyma.18G091500	GmADH-like	GCACCTCTACAAGGCGAATC	GTCGGCAAGTTTACACACGA
Glyma.12G020500	GmUKN1	TGGTGCTGCCGCTATTTACTG	GGTGGAAGGAACTGCTAACAATC
Glyma.06G038500	GmUKN2	GCCTCTGGATACCTGCTCAAG	ACCTCCTCCTCAAACTCCTCTG
-	M13-pUC	GTTTTCCCAGTCACGAC	CAGGAAACAGCTATGAC
