# Supplementary material for: Root-enriched β-amylase GmBAM-like 1 enhances drought and salt tolerance in Arabidopsis
Source: Genet Mol Biol. 2026 Aug 14;49(Suppl 3):e20250234. doi: 10.1590/1678-4685-GMB-2025-0234 (PMC13544623; doi:10.1590/1678-4685-GMB-2025-0234)
Supplement: Supplementary file 2 [file 1415-4757-GMB-49-s3-e20250234-s2.pdf]

Genetics and Molecular Biology
https://doi.org/10.1590/1678-4685-GMB-2025-0234

Supplementary Material to "Root-enriched β-amylase *GmBAM1* enhances drought and salt tolerance in Arabidopsis"

Table S2 - Gene codes for the soybean genes.

Phytozome Code	Abbreviated Code	Putative Arabidopsis homolog
Glyma.03G137900	GMRS2-like	At3g57520
Glyma.11G097300	GmSKS9-like 1	At4g38420
Glyma.06G019800	GmSKS9-like 2	
Glyma.04G236900	GmGLT1-like 1	At5g53460
Glyma.14G162300	GmGLT1-like 2	
Glyma.19G065600	GmGLT1-like 3	
Glyma.20G094500	GmGOLS2-like 1	At1g56600
Glyma.03G222000	GmGOLS2-like 2	
Glyma.19G219100	GmGOLS2-like3	
Glyma.06G007500	GmADC2-like	At4g34710
Glyma.09G168300	GmBAM-like 1	At3g23920
Glyma.16G217900	GmBAM-like 2	
Glyma.13G115500	GmLKR/SDH-like 1	At4g33150
Glyma.17G044300	GmLKR/SDH-like 2	
Glyma.18G043600	GmGAD1-like	At5g17330
Glyma.13G319000	GmADT5-like	At5g22630
Glyma.02G250800	GmMPG1-like	At4g30570
Glyma.19G043000	GmPRODH-like	At5g38710
Glyma.11G035200	GmACX1-like	At4g16760
Glyma.08G017800	GmMDAR2-like 1	At5g03630
Glyma.18G091500	GmADH-like	At5g19440
