# Supplementary material for: Root-enriched β-amylase GmBAM-like 1 enhances drought and salt tolerance in Arabidopsis
Source: Genet Mol Biol. 2026 Aug 14;49(Suppl 3):e20250234. doi: 10.1590/1678-4685-GMB-2025-0234 (PMC13544623; doi:10.1590/1678-4685-GMB-2025-0234)
Supplement: Supplementary file 3 [file 1415-4757-GMB-49-s3-e20250234-s3.pdf]

Genetics and Molecular Biology
https://doi.org/10.1590/1678-4685-GMB-2025-0234

Supplementary Material to "Root-enriched β-amylase *GmBAM1* enhances drought and salt tolerance in Arabidopsis"

Table S3 - Principal Component (PC) loadings of the Pot-Based System (PSys). The PC loadings of Figure 3 illustrate the coordinates of each variable in the biplot.

	PC1	PC2	PC3	PC4	PC5	PC6	PC7	PC8	PC9	PC10	PC11	PC12	PC13
**A. GmGOLS2-like 3**	0.017743	0.36683	-0.1315	0.77102	-0.05512	0.25354	-0.01976	0.24645	-0.12829	0.21486	-0.11106	0.18604	-0.1239
**B. GmADC2-like**	0.098996	0.097114	-0.09949	-0.33113	-0.16523	-0.18605	0.016126	0.82923	0.14257	0.2419	0.13049	0.024325	-0.12231
**C. GmBAM-like 1**	-0.02883	0.31003	-0.30219	-0.05107	-0.21445	-0.25636	0.48752	-0.20618	-0.40187	0.040811	0.22777	-0.36394	-0.26408
**D. GmGLT1-like 1**	0.10472	0.19452	0.15577	-0.36631	-0.50735	0.40237	-0.35023	-0.10282	-0.38634	-0.00909	-0.17927	0.14067	-0.19734
**E. GmBAM-like 2**	0.020665	-0.00412	-0.21397	-0.01795	0.022564	0.024692	0.093597	0.28238	-0.21295	-0.36946	-0.66551	-0.35614	0.33503
**F. GmGAD1-like**	0.071108	-0.04302	-0.08319	0.11446	-0.19782	0.24776	-0.10862	-0.02906	0.5665	-0.24298	-0.02644	-0.49278	-0.48624
**G. GmPRODH-like**	0.42178	0.34089	0.74512	0.07744	-0.03749	-0.17195	0.23004	-0.0032	0.10615	-0.00015	-0.09788	-0.17189	0.10198
**H. GmGLT1-like 2**	0.84574	-0.33128	-0.28917	0.11948	-0.01517	-0.12889	-0.15977	-0.07962	-0.14437	0.053818	0.067092	0.011653	0.000988
**I. GmGOLS2-like 2**	0.011028	0.55973	-0.25787	-0.10311	0.13031	-0.42273	-0.51059	-0.22905	0.2233	0.11176	-0.1788	-0.0283	0.07398
**J. GmGOLS2-like 1**	0.17634	0.34374	-0.11999	-0.08096	0.18985	0.10889	0.096022	0.099035	0.031751	-0.73485	0.29443	0.36599	-0.035
**K. GmMPG1-like**	-0.01124	0.07889	-0.14671	0.095008	-0.56699	0.11925	0.013169	-0.05813	0.23742	-0.01832	0.32185	-0.07645	0.67654
**L. GmRS2-like**	0.1996	0.23737	-0.16243	-0.29154	0.43746	0.59812	0.19237	-0.06963	0.097306	0.36124	0.046618	-0.17811	0.16615
**M. GmGLT1-like 3**	0.090814	-0.01427	-0.1921	-0.1191	-0.24315	-0.05274	0.4826	-0.20281	0.37064	0.10871	-0.45223	0.49081	-0.09959
