# Supplementary material for: Root-enriched β-amylase GmBAM-like 1 enhances drought and salt tolerance in Arabidopsis
Source: Genet Mol Biol. 2026 Aug 14;49(Suppl 3):e20250234. doi: 10.1590/1678-4685-GMB-2025-0234 (PMC13544623; doi:10.1590/1678-4685-GMB-2025-0234)
Supplement: Supplementary file 4 [file 1415-4757-GMB-49-s3-e20250234-s4.pdf]

Genetics and Molecular Biology
https://doi.org/10.1590/1678-4685-GMB-2025-0234

Supplementary Material to "Root-enriched β-amylase *GmBAM1* enhances drought and salt tolerance in Arabidopsis"

Table S4 - Principal Component (PC) loadings of the Hydroponics-Based System (HSys). The PC loadings of Figure 4 illustrate the coordinates of each variable in the biplot.

	PC1	PC2	PC3	PC4	PC5	PC6	PC7	PC8	PC9	PC10	PC11	PC12	PC13
**A. GmGOLS2-like 3**	0.051462	-0.60341	0.007412	-0.02551	-0.26874	0.19595	-0.59022	0.20875	-0.30339	0.035618	-0.11242	-0.0285	0.15239
**B. GmADC2-like**	-0.15344	-0.06961	0.024324	0.16882	0.10502	-0.7739	-0.44927	-0.24166	0.16319	-0.0306	0.12125	0.17109	0.028101
**C. GmBAM-like 1**	-0.34025	-0.14796	0.41943	-0.34345	-0.13698	0.089359	0.075975	0.13568	0.19528	0.30677	0.2247	0.53779	-0.2135
**D. GmGLT1-like 1**	-0.03057	-0.07273	0.21135	0.25311	0.37634	0.31189	-0.24556	-0.28972	0.025311	0.31413	0.36	-0.37426	-0.36457
**E. GmBAM-like 2**	-0.2272	-0.16238	0.28182	-0.2753	-0.06149	-0.03725	0.04282	-0.415	0.003428	-0.28424	-0.57667	-0.23103	-0.34764
**F. GmGAD1-like**	-0.01662	-0.19817	0.11274	-0.08639	0.14547	0.12235	-0.07114	0.29151	0.59091	-0.59894	0.24555	-0.19618	0.086217
**G. GmPRODH-like**	-0.28316	-0.12972	0.31368	0.79299	-0.20248	0.053584	0.24603	0.092632	0.079925	-0.02259	-0.19218	0.044273	0.13038
**H. GmGLT1-like 2**	0.6571	0.28518	0.64587	0.020784	-0.18503	-0.05616	-0.15406	-0.00612	-0.01566	-0.06973	-0.0148	0.048995	0.020375
**I. GmGOLS2-like 2**	0.3988	-0.64091	-0.01321	-0.02128	-0.02812	-0.24621	0.51548	-0.20932	-0.01117	0.077066	0.22333	-0.05084	-0.01018
**J. GmGOLS2-like 1**	-0.36268	0.12253	0.28701	-0.20457	-0.22969	-0.1847	0.12392	0.008046	-0.37733	-0.06165	0.39854	-0.46015	0.33194
**K. GmMPG1-like**	-0.04692	-0.04381	0.094855	-0.01838	0.29234	0.32367	-0.01348	-0.53362	-0.15576	-0.26319	0.083507	0.40031	0.50064
**L. GmRS2-like**	0.005431	-0.06738	0.18776	-0.18125	0.36792	-0.07837	0.022675	0.11758	0.28732	0.48922	-0.37494	-0.2291	0.50647
**M. GmGLT1-like 3**	-0.02077	-0.0885	0.21045	0.028537	0.6179	-0.17149	0.10396	0.43291	-0.48725	-0.20438	-0.0795	0.13172	-0.18535
