# Supplementary material for: Root-enriched β-amylase GmBAM-like 1 enhances drought and salt tolerance in Arabidopsis
Source: Genet Mol Biol. 2026 Aug 14;49(Suppl 3):e20250234. doi: 10.1590/1678-4685-GMB-2025-0234 (PMC13544623; doi:10.1590/1678-4685-GMB-2025-0234)
Supplement: Supplementary file 5 [file 1415-4757-GMB-49-s3-e20250234-s5.pdf]

Genetics and Molecular Biology
https://doi.org/10.1590/1678-4685-GMB-2025-0234

Supplementary Material to "Root-enriched β-amylase *GmBAM1* enhances drought and salt tolerance in Arabidopsis"

Table S5 - Genes used in phylogenetic analysis, with species specifications and codes.

Specie	*Phytozome Code*	*Abbreviated Code*
Ananas comosus	Aco011639.1	AcBAM3.1
Ananas comosus	Aco014607.1	AcBAM1
Ananas comosus	Aco009314.1	-
Ananas comosus	Aco004465.1	AcBAM10
Ananas comosus	Aco027698.1	-
Ananas comosus	Aco009092.1	-
Ananas comosus	Aco013168.1	AcBAM8
Ananas comosus	Aco001939.1	AcBAM9.1
Ananas comosus	Aco030337.1	AcBAM9.2
Ananas comosus	Aco001937.1	AcBAM9.3
Ananas comosus	Aco022314.1	AcBAM3.2
Ananas comosus	Aco030335.1	AcBAM9.4
Ananas comosus	Aco027697.1	-
Arabidopsis thaliana	AT4G17090.1	AtBAM3
Arabidopsis thaliana	AT3G23920.1	AtBAM1
Arabidopsis thaliana	AT4G15210.1	AtBAM5
Arabidopsis thaliana	AT2G32290.1	AtBAM6
Arabidopsis thaliana	AT4G00490.1	AtBAM2
Arabidopsis thaliana	AT2G45880.1	AtBAM7
Arabidopsis thaliana	AT5G55700.1	AtBAM4
Arabidopsis thaliana	AT5G45300.3	AtBAM8
Arabidopsis thaliana	AT5G18670.1	AtBAM9
Glycine max	Glyma.01G203400.1	GmBAM3.1
Glycine max	Glyma.05G068000.1	GmBAM3.2
Glycine max	Glyma.11G039400.1	GmBAM3.3
Glycine max	Glyma.17G150100.1	GmBAM3.4
Glycine max	Glyma.09G168300.1	GmBAM1.1
Glycine max	Glyma.16G217900.1	GmBAM1.2
Glycine max	Glyma.06G301500.1	-
Glycine max	Glyma.12G102900.1	-
Glycine max	Glyma.12G197100.1	-
Glycine max	Glyma.13G305200.1	-
Glycine max	Glyma.18G232400.1	GmBAM7.1
Glycine max	Glyma.09G260100.1	GmBAM7.2
Glycine max	Glyma.05G219200.1	GmBAM10.1
Glycine max	Glyma.08G025500.1	GmBAM10.2
Glycine max	Glyma.09G217000.1	GmBAM8.1

Specie	*Phytozome Code*	*Abbreviated Code*
Glycine max	Glyma.01G151800.1	GmBAM8.2
Glycine max	Glyma.13G305300.2	-
Glycine max	Glyma.15G098100.1	GmBAM9.1
Glycine max	Glyma.13G215000.1	GmBAM9.2
Oryza sativa	LOC_Os10g41550.1	OsBAM3
Oryza sativa	LOC_Os03g04770.1	OsBAM1.1
Oryza sativa	LOC_Os10g32810.1	OsBAM1.2
Oryza sativa	LOC_Os07g35940.1	-
Oryza sativa	LOC_Os09g39570.1	-
Oryza sativa	LOC_Os07g35880.1	-
Oryza sativa	LOC_Os01g13550.1	OsBAM10
Oryza sativa	LOC_Os02g03690.1	OsBAM8
Oryza sativa	LOC_Os07g47120.1	OsBAM9.1
Oryza sativa	LOC_Os03g22790.1	OsBAM9.2
Oryza sativa	LOC_Os01g70700.1	-
Populus trichocarpa	Potri.001G148900.2	PtBAM3.1
Populus trichocarpa	Potri.003G085500.1	PtBAM3.2
Populus trichocarpa	Potri.010G062900.1	PtBAM1.1
Populus trichocarpa	Potri.008G174100.1	PtBAM1.2
Populus trichocarpa	Potri.017G040800.7	-
Populus trichocarpa	Potri.014G083800.1	-
Populus trichocarpa	Potri.014G083700.6	PtBAM7.1
Populus trichocarpa	Potri.002G159300.16	PtBAM7.2
Populus trichocarpa	Potri.003G143500.1	PtBAM10.1
Populus trichocarpa	Potri.001G087600.1	PtBAM10.2
Populus trichocarpa	Potri.003G106500.6	PtBAM8
Populus trichocarpa	Potri.008G204200.1	PtBAM9
Setaria viridis	Sevir.9G323200.3	SvBAM3
Setaria viridis	Sevir.9G549100.1	SvBAM1.1
Setaria viridis	Sevir.9G229200.1	SvBAM1.2
Setaria viridis	Sevir.2G353100.1	-
Setaria viridis	Sevir.2G140000.2	-
Setaria viridis	Sevir.2G353000.1	-
Setaria viridis	Sevir.5G004100.4	SvBAM10
Setaria viridis	Sevir.1G099050.1	SvBAM8
Setaria viridis	Sevir.2G435300.1	SvBAM9.1
Setaria viridis	Sevir.9G407700.1	SvBAM9.2
Solanum lycopersicum	Solyc08g007130.4.1	SlBAM3.1
Solanum lycopersicum	Solyc08g077530.3.1	SlBAM3.2
Solanum lycopersicum	Solyc09g091030.3.1	SlBAM1
Solanum lycopersicum	Solyc01g094580.3.1	SlBAM7
Solanum lycopersicum	Solyc08g082810.4.1	SlBAM10
Solanum lycopersicum	Solyc08g005780.4.1	SlBAM8
Solanum lycopersicum	Solyc01g067660.3.1	SlBAM9
Solanum lycopersicum	Solyc07g052690.4.1	-
Vitis vinifera	VIT_202s0012g00170.1	VvBAM3
Vitis vinifera	VIT_205s0051g00010.2	VvBAM1.1

Specie	***Phytozome Code***	***Abbreviated Code***
Vitis vinifera	VIT_205s0077g00280.1	VvBAM1.2
Vitis vinifera	VIT_212s0059g02670.1	-
Vitis vinifera	VIT_215s0046g02620.1	-
Vitis vinifera	VIT_215s0046g02640.1	VvBAM7
Vitis vinifera	VIT_202s0025g02120.1	VvBAM10
Vitis vinifera	VIT_219s0015g00500.1	VvBAM4
Vitis vinifera	VIT_202s0087g00430.1	VvBAM8
Vitis vinifera	VIT_205s0020g01910.1	VvBAM9
