# Supplementary material for: Root-enriched β-amylase GmBAM-like 1 enhances drought and salt tolerance in Arabidopsis
Source: Genet Mol Biol. 2026 Aug 14;49(Suppl 3):e20250234. doi: 10.1590/1678-4685-GMB-2025-0234 (PMC13544623; doi:10.1590/1678-4685-GMB-2025-0234)
Supplement: Supplementary file 6 [file 1415-4757-GMB-49-s3-e20250234-s6.pdf]

Genetics and Molecular Biology
https://doi.org/10.1590/1678-4685-GMB-2025-0234

Supplementary Material to "Root-enriched β-amylase *GmBAM1* enhances drought and salt tolerance in Arabidopsis"

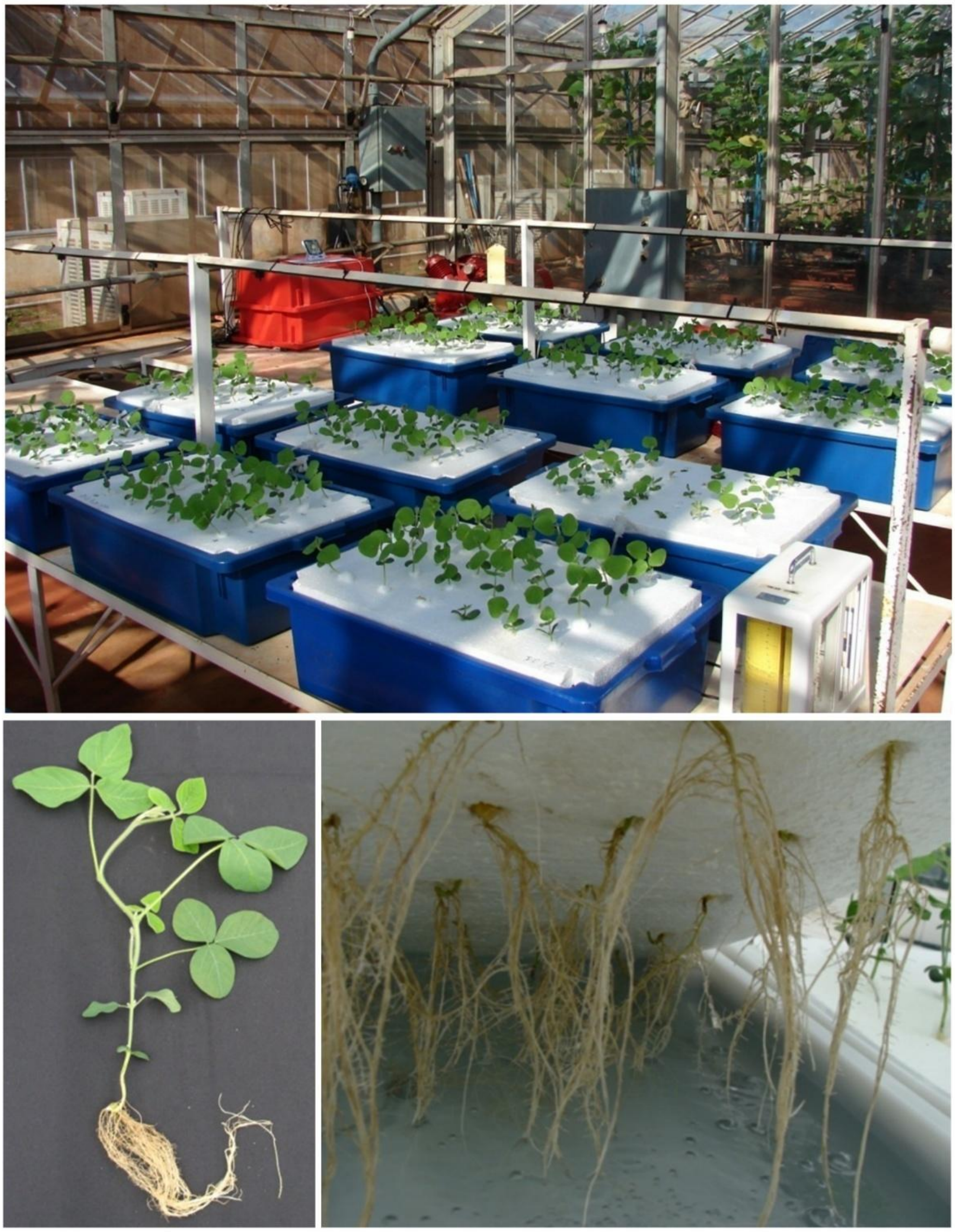

Figure S1 - Soybean plants growing in the hydroponic system**.** Bottom images show the details of a single plant and the development of the roots. Images of the greenhouse with the hydroponic system were provided by Prof. Alexandre Lima Nepomuceno.
