# Supplementary material for: Root-enriched β-amylase GmBAM-like 1 enhances drought and salt tolerance in Arabidopsis
Source: Genet Mol Biol. 2026 Aug 14;49(Suppl 3):e20250234. doi: 10.1590/1678-4685-GMB-2025-0234 (PMC13544623; doi:10.1590/1678-4685-GMB-2025-0234)
Supplement: Supplementary file 7 [file 1415-4757-GMB-49-s3-e20250234-s7.pdf]

Genetics and Molecular Biology
https://doi.org/10.1590/1678-4685-GMB-2025-0234

Supplementary Material to "Root-enriched β-amylase *GmBAM1* enhances drought and salt tolerance in Arabidopsis"

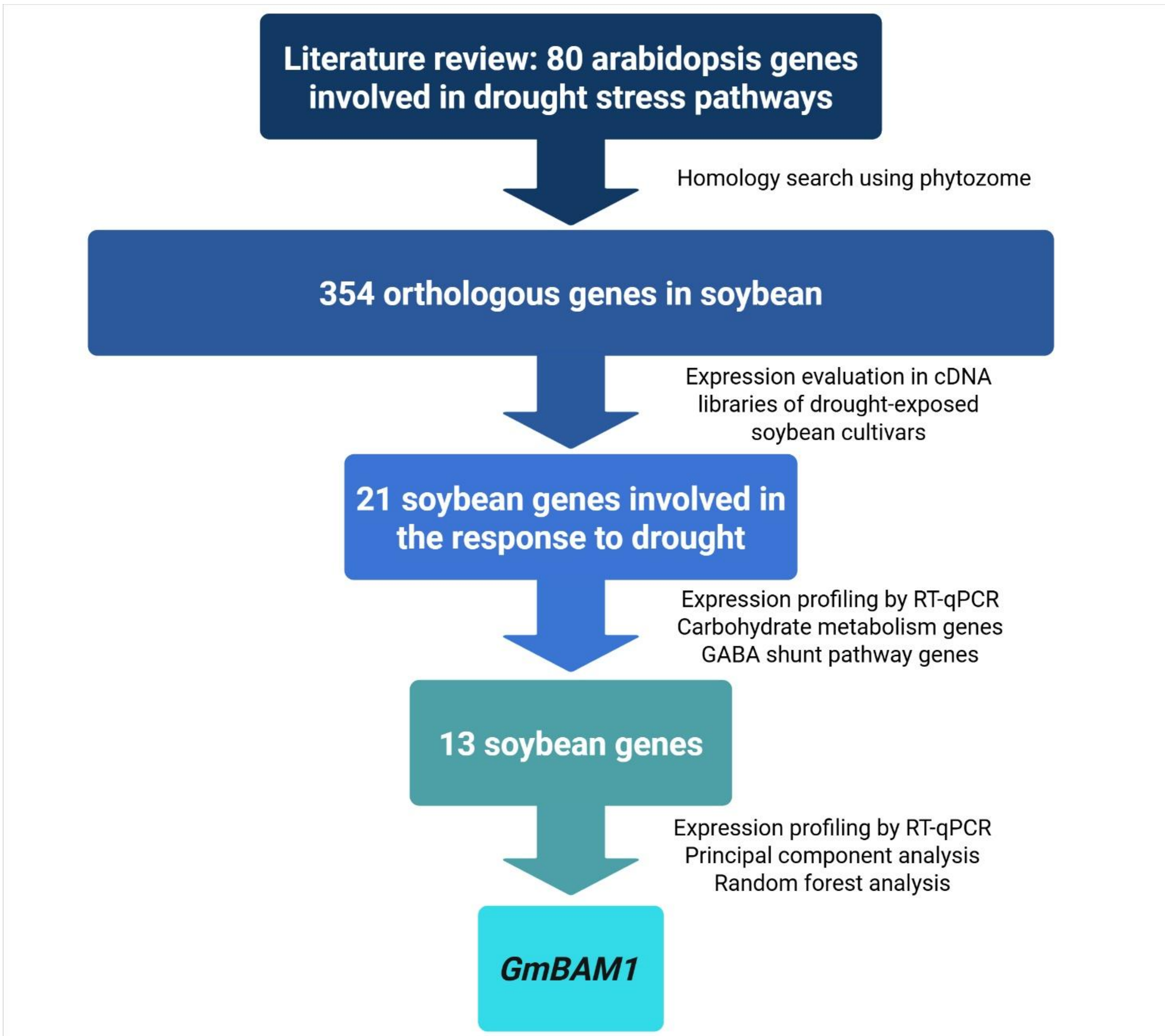

Figure S2 - Gene selection strategy. Flowchart detailing the gene selection strategy. Candidate genes were initially identified based on a literature review and soybean orthologs were identified using the Phytozome database. Subsequent filtering steps included expression profiling and restricting the list to specific metabolic pathways. The selection of *GmBAM1* involved the evaluation of gene expression and the application of multivariate analyses. Image created in https://BioRender.com.
