# Supplementary material for: Root-enriched β-amylase GmBAM-like 1 enhances drought and salt tolerance in Arabidopsis
Source: Genet Mol Biol. 2026 Aug 14;49(Suppl 3):e20250234. doi: 10.1590/1678-4685-GMB-2025-0234 (PMC13544623; doi:10.1590/1678-4685-GMB-2025-0234)
Supplement: Supplementary file 8 [file 1415-4757-GMB-49-s3-e20250234-s8.pdf]

Genetics and Molecular Biology
https://doi.org/10.1590/1678-4685-GMB-2025-0234

Supplementary Material to "Root-enriched β-amylase *GmBAM1* enhances drought and salt tolerance in Arabidopsis"

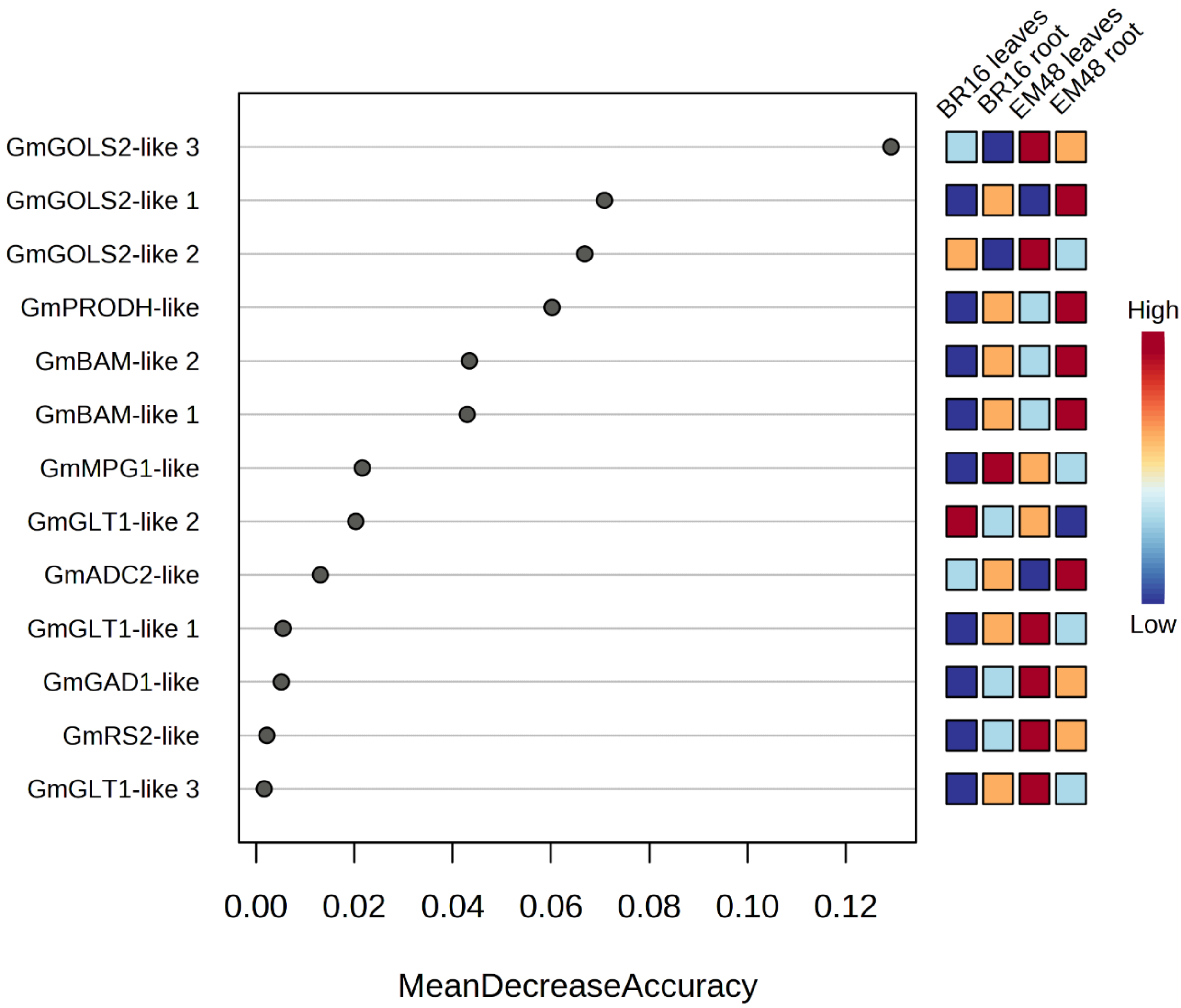

Figure S3 - Random Forest analysis of gene importance in the hydroponic system (HSys). Variable importance plot showing the ranking of the 13 genes based on mean decrease in classification accuracy when the variable is permuted. Random Forest classification was performed on $\log_{10}$-transformed NRQs (see Methods). Higher mean decrease in accuracy indicates greater importance for discriminating drought response among samples. Analysis was performed in R using the randomForest package with parameters as reported in Materials and Methods.
