# Supplementary material for: Root-enriched β-amylase GmBAM-like 1 enhances drought and salt tolerance in Arabidopsis
Source: Genet Mol Biol. 2026 Aug 14;49(Suppl 3):e20250234. doi: 10.1590/1678-4685-GMB-2025-0234 (PMC13544623; doi:10.1590/1678-4685-GMB-2025-0234)
Supplement: Supplementary file 9 [file 1415-4757-GMB-49-s3-e20250234-s9.pdf]

Genetics and Molecular Biology
https://doi.org/10.1590/1678-4685-GMB-2025-0234

Supplementary Material to "Root-enriched β-amylase *GmBAM1* enhances drought and salt tolerance in Arabidopsis"

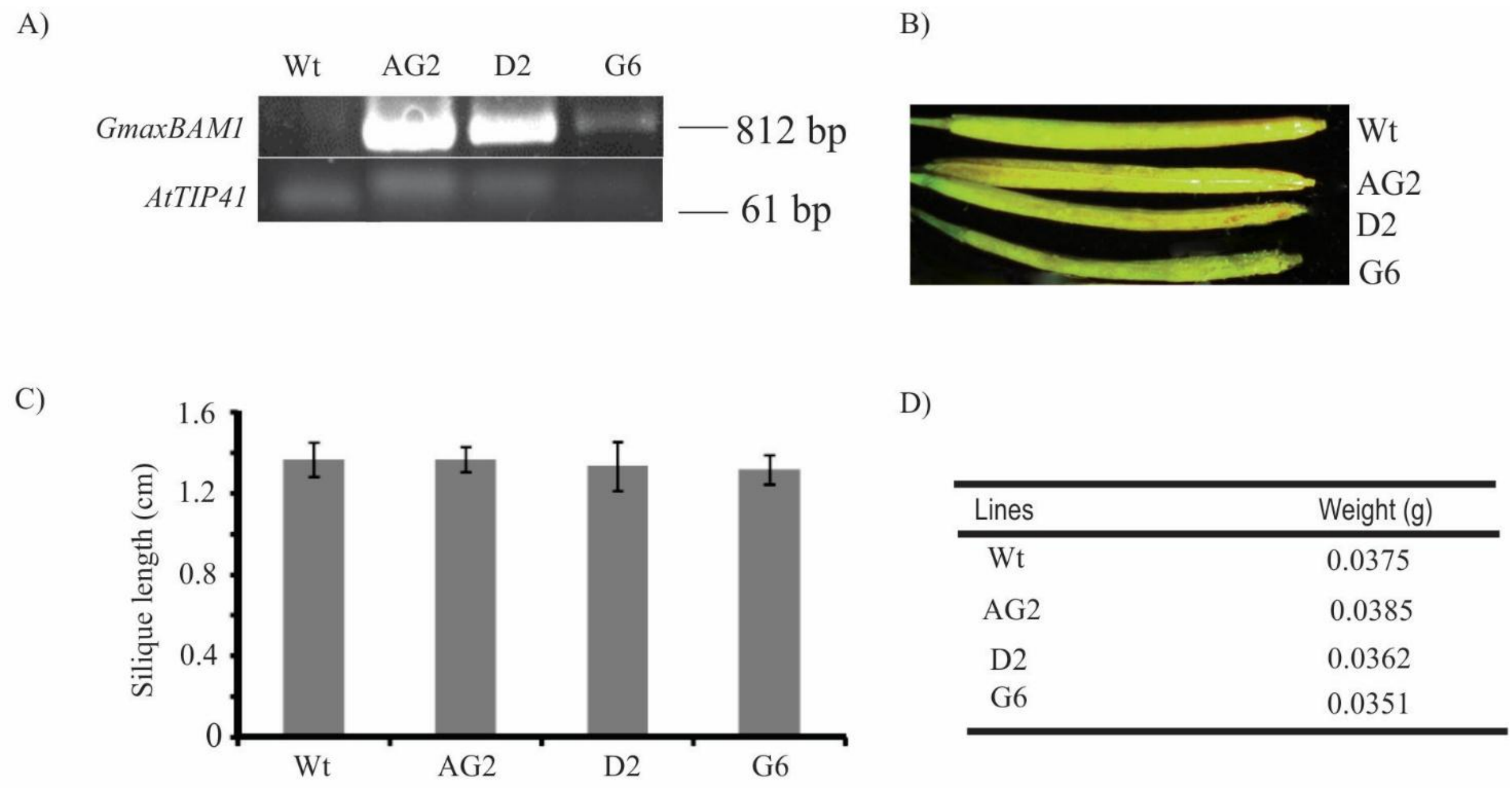

Lines	Weight (g)
Wt	0.0375
AG2	0.0385
D2	0.0362
G6	0.0351

Figure S4 - Confirmation of transgene expression and morphological comparison between *GmBAM1*-overexpressing lines and WT. A. RT-PCR verification of *GmBAM1* expression in independent transgenic lines (AG2, D2, G6) and WT (negative control). Primers and cycling conditions are listed in Materials and Methods. A representative gel image is shown. B. Representative siliques from WT and transgenic plants (photograph). C. Silique length measured on the fourth and fifth siliques of each plant (n = 20 siliques per genotype; mean ± SE). D. Seed weight measured for siliques indicated in (C) (n = 40 seeds per genotype; mean ± SE). Statistical comparison by Student's t-test showed no significant difference between WT and transgenic lines ($P < 0.05$).
